# A systematic review of clinical and diagnostic findings, treatment, and outcomes in canine neosporosis cases evaluated by magnetic resonance imaging

**DOI:** 10.3389/fvets.2025.1669529

**Published:** 2025-10-22

**Authors:** Neringa Alisauskaite

**Affiliations:** Northwest Veterinary Specialists Part of Linnaeus Group, Runcorn, United Kingdom

**Keywords:** canine, neosporosis, dog, neospora, magnetic resonance imaging, CNS infection, central nervous system, MRI

## Abstract

**Background:**

While magnetic resonance imaging (MRI) is increasingly used in the diagnostic workup of canine central nervous system (CNS) disorders, its role in neosporosis has not been systematically characterised. Numerous questions remain regarding optimal diagnostic approaches and treatment protocols, and large-scale studies are lacking.

**Objectives:**

The objective of the study was to systematically review and analyse published cases of canine neosporosis undergoing MRI examination of the CNS and identify clinical, diagnostic, and treatment variables associated with outcomes.

**Methods:**

A comprehensive literature search was performed using PubMed and Google Scholar. The inclusion criteria were confirmation of *N. caninum* infection by serology, polymerase chain reaction (PCR), or histopathology, and MRI performed at diagnosis. Extracted data included signalment, clinical signs, MRI features, cerebrospinal fluid (CSF) results, serology, PCR, treatment, and outcomes. Statistical analyses assessed associations and correlations among diagnostic and outcome variables.

**Results:**

Eighty-two cases extracted from 11 publications met the inclusion criteria. MRI commonly revealed multifocal intra-axial lesions, frequently involving the cerebellum, with variable contrast enhancement of the brain and spinal cord and meninges, and consistent contrast enhancement in the muscles. A significant association was identified between elevated cerebrospinal fluid total nucleated cell count (CSF TNCC) and positive cerebrospinal fluid polymerase chain reaction (CSF PCR). Treatment duration strongly correlated with survival. Death/euthanasia due to neosporosis occurred in one-third of the cases. Remissions were rare. Relapses occurred despite initial improvement.

**Conclusion:**

Canine neosporosis presents with diverse neurological clinical signs and MRI features. The disease mainly affects young-to-middle-aged adults with multifocal CNS involvement, and CSF is abnormal in the majority of cases. Review analyses showed that serology titres, serum creatine kinase (CK) levels, CSF TNCC, CSF PCR results, and MRI contrast had no prognostic value, while higher CSF TNCC was associated with positive CSF PCR. The only consistent prognostic marker was treatment duration—longer antimicrobial therapy was correlated with improved survival. Although the prognosis remains guarded, early recognition and prolonged antimicrobial treatment are key to improving outcomes.

## Introduction

*Neospora caninum* is a protozoan parasite and a significant cause of neuromuscular and central nervous system (CNS) disease in dogs (1). Although traditionally regarded as a condition of young puppies, adult-onset neosporosis is increasingly recognized and may present with a wide range of neurological signs, including cerebellar dysfunction, spinal cord involvement, and neuromuscular deficits (1–4). Neosporosis remains a rare canine infectious disease, with a prevalence of 2.25% among CNS inflammatory diseases in the United Kingdom (5).

The diagnosis of neosporosis typically involves a combination of clinical suspicion, serological testing, cerebrospinal fluid (CSF) analysis, and polymerase chain reaction (PCR) detection of *N. caninum* DNA (1). Magnetic resonance imaging (MRI) has become an essential tool in the evaluation of canine neurological disorders, yet its role in the diagnosis and characterisation of neosporosis remains underexplored (2, 3, 6, 7). Existing reports describe findings such as cerebellar atrophy, intra-axial lesions, meningeal enhancement, and muscle involvement, but these are limited to case reports and a few larger studies (2–4, 6–8).

This study aimed to systematically review all published cases of canine neosporosis undergoing MRI of the CNS, compiling clinical, diagnostic, imaging, therapeutic, and outcome data from canine neosporosis—the largest such cohort described to date. In addition to providing descriptive analysis, we formulated and tested by performing various statistical tests, several hypotheses regarding diagnostic and prognostic indicators:

1. Higher *N. caninum* serology titres would be associated with higher CSF total nucleated cell count (TNCC).
2. Positive CSF PCR results would be associated with higher CSF TNCC.
3. Higher serology titres would be associated with positive CSF PCR results.
4. Higher serology titres, higher serum CK values, and positive CSF PCR findings would be associated with worse clinical outcomes.
5. Corticosteroid use as part of the treatment protocol would correlate with poorer clinical outcomes.
6. Longer durations of antimicrobial therapy would correlate with improved survival.
7. Contrast enhancement in MRI studies will be associated with shorter survival.

## Materials and methods

This study was conducted as a systematic review of published literature describing clinical cases (extracted from case reports, case series, and clinical studies) of canine neosporosis with confirmed CNS involvement supported by MRI. A comprehensive search of two electronic databases—PubMed and Google Scholar—was performed. The search strategy employed combinations of the keywords ‘canine’, ‘neosporosis’, ‘dog’, ‘neospora’, ‘magnetic resonance imaging’, ‘CNS’, ‘central nervous system’, and ‘MRI’. In addition, reference lists of identified articles were manually reviewed to capture additional relevant publications not retrieved through electronic searching.

### Inclusion criteria

Cases were included if they had a confirmed diagnosis of *Neospora caninum* infection by at least one of the following: positive serological titre (≥1:800), detection of *N. caninum* DNA via PCR from CSF or tissue, or histopathological confirmation (with or without immunohistochemistry or PCR confirmation), with MRI of the CNS (brain and/or spinal cord) performed at diagnosis including at least a basic description of findings, neurological localisation determined at presentation, and available information on treatment protocols.

### Data extraction

From each study, the following data were extracted and entered into a standardised spreadsheet:

- Signalment and epidemiological data: age, breed, sex, neuter status, and geographical origin.
- Clinical presentation: duration of clinical signs, presenting complaints, neurological examination findings, and neurological localisation, as described by the authors.

### Diagnostic investigations

#### MRI Findings

All included cases underwent MRI of the CNS, and in some cases, the appearance of adjacent musculature and peripheral structures was also assessed, where such descriptions were available. Special attention was drawn to lesion distribution (brain/spinal cord; grey/white matter), contrast enhancement patterns (intraparenchymal, meningeal, and intramuscular), presence of cerebellar abnormalities (particularly atrophy and T2-weighted (T2W)/Fluid-Attenuated Inversion Recovery (FLAIR) hyperintense cerebellar rim changes), and involvement of the masticatory or paraspinal muscles.

#### CSF analysis and cytology categorisation

Total nucleated cell count, protein concentration, and cytology results were collected where available. Protein concentrations in CSF above 0.3 g/L for the cisternal tap and above 0.45 g/L for the lumbar tap were considered abnormal. Normal CSF cell count with elevated protein levels was regarded as albuminocytological dissociation (ACD). Pleocytosis was defined as TNCC > 5 cells/μL for cisternal and lumbar taps. CSF cytology was categorised as follows based on descriptions of De Lorenzi et al. (9):

- Mononuclear pleocytosis: predominance of mononuclear cells (>70% lymphocytes and/or monocytes/macrophages).
- Neutrophilic pleocytosis: presence of neutrophils exceeding 25%.
- Mixed pleocytosis: no clear predominance of one cell type.
- Eosinophilic pleocytosis: presence of eosinophils exceeding 1%.

In some cases, the classification used by the authors was different from the classification described by Lorenzi et al. In order to have a unified classification of CSF cytology findings and allow attempts for statistical analysis for the collected cases, we reclassified the cytology results where necessary based on descriptions provided by the authors and adapted them to the Lorenzi et al. cytology classification. In cases where exact cell distribution in cytology samples was not provided, qualitative descriptions from the original reports (e.g., ‘predominantly neutrophils’, ‘large neutrophilic component’, or ‘presence of eosinophils’) were used to classify cytology in cases where neutrophilic or eosinophilic pleocytosis was highly suspected to be present. When both neutrophils and eosinophils were present in cytology samples, the presence of eosinophils took precedence in classification. This standardised approach ensured uniformity across heterogeneous reporting formats.

#### Serum biochemistry

Serum biochemistry abnormalities as reported by the authors, with particular emphasis on creatine kinase (CK) and aspartate aminotransferase (AST) concentrations, were recorded when available.

#### Serology

Indirect fluorescent antibody (IFA) titres were documented; follow-up titres at particular time points after diagnosis were included if available.

#### CSF PCR

Results from PCR testing for *N. caninum* were noted, where available, as positive and negative. Cycle thresholds were not reported individually to allow for simplification in reporting.

When complete data were not available for individual cases, the total denominator was adjusted accordingly for descriptive statistical analysis.

### Treatment and outcome measures

Treatment protocols varied across the included studies and were recorded individually for each case, as described by the authors. Therapeutic data were extracted when available and included the names of medications administered, combinations of drugs used, and total duration of treatment with antimicrobials expressed in months. In cases where treatment was ongoing at the time point of the last follow-up, this was noted and, for statistical analysis, was defined as ‘>’ than the follow-up time (in months) for the particular case (e.g., ‘>8’). Treatment with corticosteroids was recorded in our collected data; however, the type of medication, length of treatment, and dosages were not specified because the data were too heterogeneous. If the antimicrobial type, other medications, or treatment duration were not specified, this was documented. These cases were excluded from statistical analyses involving corticosteroid use and treatment duration but were included in the descriptive summaries. The duration for each antimicrobial medication and treatment modification by replacing or adding new antimicrobial medications were not individually described because of the heterogeneity and in many instances, the lack of detailed description of the data.

Clinical follow-up and outcome data were obtained from published cases. For each case, the presence or absence of reported outcome data, the total duration of clinical follow-up (in months), and the survival time from diagnosis to death or last follow-up (in months) were recorded. If follow-up duration in publications was reported as a range (e.g., 8–12 weeks) for all cases, the shortest duration in the range (e.g., 8 weeks, converted to months) was used for data collection and subsequent analysis in our review.

When outcome data were available, the clinical status at the last follow-up was categorised into one of the following:

(1) Remission (defined as full resolution of neurological signs),
(2) Clinical improvement (defined as improvement of neurological signs with residual neurological deficits),
(3) Relapse following improvement/remission,
(4) Euthanasia or death due to *Neospora caninum*-associated neurological signs,
(5) Euthanasia or death due to causes unrelated to neosporosis (e.g., comorbidities or non-neurological complications), or,
(6) Euthanasia based on owner decision before initiation of treatment.

The occurrence of relapses after initial improvement/remission was documented, including the time (in months) from diagnosis to relapse. Where applicable and available, the time from diagnosis to euthanasia or death was recorded in months.

The time intervals between diagnosis and death/euthanasia, improvement and relapse, and total treatment duration were systematically recorded in months where possible. Dogs that were lost to follow-up were excluded from survival analyses but were included in descriptive summaries where appropriate.

### Data analysis

Descriptive statistics (prevalence, means, medians, and ranges) were used to summarise data. Percentages were calculated for categorical variables, and continuous variables were described using median and mean values where appropriate. Comparative analysis was performed across cohorts and treatment categories to identify patterns in signalment, history, examination, investigation findings, imaging findings, therapies, and clinical outcomes.

### Statistical analysis

The Kruskal–Wallis test, adjusted for ties, was used to assess the relation between *Neospora caninum* serology titres and CSF TNCC. Serology titres were categorised into three groups of variables—serology titres under 1:800, specified as equal to 1:800, and above 1:800. If the results of the publication were stating that the serology titre was ≥1:800, it was removed from statistical calculations. Serology titre groups were compared to CSF TNCC as continuous parameters.

The Mann–Whitney U-tests, adjusted for ties, were used to calculate the association between CSF PCR results and CSF TNCC. CSF TNCC was used as a continuous variable, and PCR results were used as two variables—positive and negative.

The Mann–Whitney U-tests, adjusted for ties, was used to calculate the association between contrast enhancement in MRI studies and survival. Survival was used as a continuous variable, and contrast enhancements in MRI were used as two variables—positive and negative.

Fisher’s exact test was used for the analysis of the relation between serology titres for *Neospora caninum* and CSF PCR findings. PCR findings were used as two categories—positive and negative. These parameters were compared to three serology titre categories. Serology titres were categorised into three groups of variables—serology titres under 1:800, specified as equal to 1:800, and above 1:800. If the results of the publication stated that the serology titre was ≥1:800, it was removed from statistical calculations.

Spearman’s rank correlation test was used to calculate the correlation between CSF TNCC and CK values at the time of diagnosis and survival. TNCC, CK values, and survival in months were used as continuous variables, both including and excluding censored survival data. In addition, the correlation between CSF TNCC and survival in months in cases of death/euthanasia due to neosporosis was assessed using the same method.

The Kaplan–Meier plots were used to assess the relation between serology titres and survival. Serology test results were categorised into three groups—serology titres under 1:800, equal to 1:800, and above 1:800, and these groups were compared to duration of survival using the log-rank test. When patients survived beyond the follow-up time point, they were regarded as censored parameters.

The Kaplan–Meier plots were used to assess the relation between CSF PCR findings and survival, where PCR from CSF test results was categorised into two groups—positive and negative, and these were compared to the duration of survival using the log-rank test. When patients survived beyond the follow-up time point, they were regarded as censored parameters.

The Kaplan–Meier plots were used for the relation between corticosteroid use for treatment of neosporosis and survival analysis. Corticosteroid use as part of the treatment protocol at any point of the treatment was categorised into two groups—used and not used, and these were compared to duration of survival using the log-rank test. When patients survived beyond the follow-up time point, they were censored parameters.

Spearman’s rank correlation test was used to calculate the correlation between two continuous (including and excluding censored data) variables—duration of antimicrobial treatment and survival. If dogs survived beyond the follow-up time, the time point of last follow up was used for analysis and marked separately in the graph compared to dogs that died/were euthanised at a known time point.

Significance was taken as *p* < 0.05. The correlation coefficient (r) was used to calculate the correlation where appropriate. All calculations were done using Minitab^®^ 21 statistical software (Pennsylvania, US).

## Results

Detailed information is available in the Supplementary materials.

### Epidemiology

Labrador Retrievers were the most commonly affected, accounting for 25.6% (21/82) of cases. Greyhounds were the next most frequently represented breed (21.9%, 18/82), followed by West Highland White Terriers and Lurchers, each comprising 7.3% (6/82). Cavalier King Charles Spaniels made up 4.9% (4/82), French Bulldogs 3.6% (3/82), and both Border Collies and Whippets 2.4% (2/82).

Geographically, the majority of cases originated from the United Kingdom (79.3%, 65/82), while 15.8% (13/82) were reported from Australia. A single case (1.2%) was reported from Spain, and the country of origin was not specified in 3.6% (3/82) of cases. However, these may have originated from Italy, Germany, the United Kingdom, Switzerland, or Finland, as suggested by the authors.

### Clinical and neurological findings

Summarised information on signalment, history, and clinical and neurological examination findings is presented in Table 1. Age at onset ranged from 0.3 to 12 years, with a mean of 4.9 years and a median of 4.5 years. Fourteen dogs (17.1%) were juveniles (≤1 year), and 26 (31.7%) were aged 2 years or younger. Twenty dogs (24.4%) were 8 years or older, while the remaining 34 dogs (41.4%) were between 3 and 7 years old. Males accounted for 61% (50/82) of cases, while females represented 39% (32/82).

**Table 1:** Summary of key clinical, diagnostic, and laboratory findings in 82 MRI-confirmed cases of canine CNS neosporosis.

Domain	Parameter	Finding
Neurological examination findings	Most common signs (*n* = 69)	Ataxia: 55.1% (38/69) Limb paresis: 49.3% (34/69) Cerebellar ataxia/hypermetria: 23.2% (16/69) Head tilt: 23.2% (16/69) Non-ambulatory: 14.5% (10/69) Tremors: 13.0% (9/69) Seizures: 7.2% (5/69)
	CNS involvement only	82.6% (57/69)
	Neuromuscular involvement only	5.8% (4/69)
	Mixed CNS + neuromuscular	11.6% (8/69)
Neurological localisation	Multifocal CNS	57.3% (47/82)
	Brain only	50.8% (33/65)
	Spinal cord only	23.1% (15/65)
	Mixed brain + spinal	7.7% (5/65)
	PNS only	7.7% (5/65)
	CNS + PNS	10.8% (7/65)
	Cerebellum affected	40% (26/65) cerebellum only 16.9% cerebellum + brainstem 9.2% cerebellum + forebrain 7.7%
CSF Findings	CSF abnormal	94.1% (64/68)
	Pleocytosis	79.4% (54/68), mean TNCC 64.1/μL (median: 17; range: 0–1,450)
	Protein concentration	Elevated in 90.5% (19/21), mean: 1.4 g/L (median: 0.85; range: 0.34–9.92)
	ACD	14.7% (10/68)
CSF Cytology	Mononuclear pleocytosis	27.9% (19/68)
	Mixed cell pleocytosis	23.5% (16/68)
	Eosinophilic pleocytosis	14.7% (10/68)
	Neutrophilic pleocytosis	11.7% (8/68)
	Normal cytology with normal TNCC	20.6% (14/68)
	ACD with normal cytology	14.7% (10/68)
	Protozoal tachyzoites observed	1 case
Serum Biochemistry	CK (*n* = 52)	Elevated in 88.5% (46/52), mean: 11,299 U/L (median: 1,747; range: 281–36,054)
	AST (*n* = 40)	Elevated in 75% (30/40), mean: 182 U/L (median: 154; range: 59–471)
	ALT (*n* = 29)	Elevated in 79.3% (23/29), mean: 1,088 U/L (median: 280; range: 95–16,815)
Serology	Available in 78/82 cases	Titres <1:800 in 5 cases =1:800 in 11 ≥1:800 in 62 (most frequent 1:800–1:1,600)
	Most common titres	1:800 (35.9%, 28/78) 1:1,600 (34.6%, 27/78) 1:3,200 (7.7%, 6/78) 1:6,400 (6.4%, 5/78)
	Follow-up serology (*n* = 14)	Decrease in 8 dogs (6 became negative/borderline), stable in 6 dogs
PCR from CSF	Tested in 51 dogs	Positive 52.9% (27/51) Negative 47.1% (24/51)

ACD, albuminocytological dissociation; ALT, alanine aminotransferase; AST, aspartate aminotransferase; CK, creatine kinase; CNS, central nervous system; CSF, cerebrospinal fluid; PCR, polymerase chain reaction; PNS, peripheral nervous system; TNCC, total nucleated cell count.

Duration of clinical signs was reported in 38 cases, ranging from 3 days to 1.5 years. The mean duration was 64.1 days, and the median was 24.5 days, suggesting that the majority of cases were diagnosed within 1 month of symptom onset. Five dogs (13.1%) presented acutely, with symptoms developing over 3–7 days. Presenting complaints were documented in 28 cases. Ataxia was the most frequent complaint reported by the owners or the referring veterinary surgeons (57.1%, 16/28), followed by paresis or paralysis of the pelvic limbs or all four limbs (42.8%, 12/28). Head tilt and urinary/faecal incontinence were each reported in 7.1% (2/28) of cases.

Physical examination findings were reported in 23 cases, with 78.3% (18/23) considered unremarkable. Abnormal findings in 5 dogs (21.7%) included pyrexia, as well as heart murmur, muscle wastage, dehydration, cachexia, and increased respiratory effort.

Neurological examination findings from the referral hospitals were available for 69 cases. CNS signs were present in 89.9% (62/69) of cases, and isolated CNS signs (without neuromuscular involvement) in 82.6% (57/69) of cases. Neuromuscular signs alone were reported in 4 dogs (5.8%), while 8 dogs (11.6%) exhibited both CNS and neuromuscular involvement.

Ataxia was documented in 38 dogs (55.1%), with cerebellar ataxia or hypermetria noted in 16 cases (23.2%). Limb paresis was observed in 34 dogs (49.3%), and 10 (14.5%) were non-ambulatory. Additional findings included seizures (7.2%, 5/69), head tilt (23.2%, 16/69), and tremors (13%, 9/69).

Neurological localisation was reported in all 82 cases. Multifocal involvement was seen in 57.3% (47/82), with 11 of these cases lacking further specification. In an additional 6 cases, the description of localisation was limited to CNS only (without specification if the brain, spinal cord, or both were affected) (in 2 cases), or CNS and peripheral nervous system (PNS) (in 4 cases) were mentioned.

Of the cases with more description of neurological localisation available, exclusive brain localisation was noted in 50.8% (33/65) of cases, and spinal cord-only localisation in 23.1% (15/65) of cases. Mixed brain and spinal cord involvement was documented in 7.7% (5/65). Based on neurological localisation descriptions, the cerebellum was affected in 40% (26/65) of cases, either alone or with other regions:

- Cerebellum only: 16.9% (11/65)
- Cerebellum and brainstem: 9.2% (6/65)
- Cerebellum and forebrain: 7.7% (5/65)

Forebrain and brainstem was localised in 9.2% (6/65) cases. PNS-only localisation was found in 7.7% (5/65), while combined CNS and PNS involvement was identified in 10.8% (7/65).

### CSF analysis

CSF analysis results are presented in Table 1, and they were unavailable or incomplete in 14 of 82 cases. Of the remaining 68 cases, pleocytosis was described without further information in 14 cases. Unremarkable TNCC and protein concentration were reported in 4 cases (5.9%), while 64 cases (94.1%) had abnormal CSF findings.

ACD was noted in 14.7% (10/68), and increased TNCC was noted in 79.4% (54/68). Exact cell counts were available in 54 cases, with a mean TNCC of 64.1 cells/μL, a median of 17 cells/μL, and a range of 0–1,450 cells/μL.

Protein concentration data were available in 21 cases. Levels were elevated in 19 cases, with a mean of 1.4 g/L, a median of 0.85 g/L, and a range of 0.34–9.92 g/L. Two cases had unremarkable protein concentrations.

### CSF cytology

CSF cytology results are presented in Table 1, and they were unavailable in 14 cases, one of which was described to present an unspecified pleocytosis in the CSF. Of the remaining 68 cases 14 cases (20.6%) had unremarkable cell count and cytology with 10 (14.7%) of them showing ACD.

Abnormal cytology was observed in 54/68 (77.9%) of cases. Among abnormal findings:

- Mononuclear pleocytosis was observed in 27.9% (19/68) of cases
- Mixed cell pleocytosis was observed in 23.5% (16/68) of cases
- Neutrophilic pleocytosis was observed in 11.7% (8/68) of cases
- Eosinophilic pleocytosis was observed in 14.7% (10/68) of cases

One case demonstrated intracellular and extracellular protozoal tachyzoites in CSF.

### Serum biochemistry abnormalities

The serum biochemistry result summary is available in Table 1.

#### Creatine kinase (CK)

CK was not measured or not reported in 36.6% (30/82) of cases. Of the 52 cases with data, 88.5% (46/52) had elevated CK, with only 11.5% (6/52) within the reference range. The mean CK was 11,299 U/L, with a median of 1,747 U/L and a range of 281–36,054 U/L. One case was excluded from this descriptive analysis due to CK exceeding measurable limits.

#### Aspartate aminotransferase (AST)

AST data were not available in 51.2% (42/82) of cases. Among 40 cases with results, 75% (30/40) cases showed elevated AST, with a mean of 182 U/L, a median of 154 U/L, and a range of 59–471 U/L. One outlier (>1,083 U/L) was excluded from the descriptive analysis.

#### Alanine aminotransferase (ALT)

ALT was unreported in 64.6% (53/82). Of the 29 reported cases, 79.3% (23/29) had elevated levels. The mean ALT was 1,088 U/L, with a median of 280 U/L and a range of 95–16,815 U/L.

### Blood serology titres

Serology and PCR results are summarised in Table 1. Among the 82 dogs diagnosed with *Neospora caninum* infection, serology titres were unavailable in 4 cases (4.9%). For the remaining 78 dogs, serology titres varied widely, ranging from 320 to 25,000, reflecting considerable heterogeneity in immune response intensity. The reporting of serology titres varied – some of them would be reported as the exact number of serology titre, and some of them as a value greater than or equal to or greater than a certain cutoff value, depending on the laboratory protocols.

The serology titres were lower than 1:800 in 5 cases. Titres were equal to 1:800 in 11 cases. In 7 cases, titres were reported to be equal to or higher than 1:800. The titres were higher than 1:800 in 55 cases.

The most frequent titres clustered approximately 1,600 (27 cases, 34.6%) and 800 (28 cases, 35.9%). Other titres included clusters 3,200 (6 cases, 7.7%), 6,400 (5 cases, 6.4%), and 12,800 (3 cases, 3.8%), with lower frequencies at 400 (3 cases, 3.8%), 600 (2 cases, 2.6%), 640 (1 case, 1.3%), 320 (1 case, 1.3%), 12,560 (1 case, 1.3%), and 25,000 (1 case, 1.3%).

Follow-up serology was available for 14 dogs, providing insight into antibody dynamics. Eight dogs exhibited declining titres over time. Among these, six initially high-positive cases demonstrated either seronegative or borderline titres (1:400) on follow-up; clinical outcomes included remission (2 dogs), improvement (2 dogs), and initial improvement followed by relapse (2 dogs). The remaining two showed titre reductions from >1:1,600 and >1:3,200 to 1:800, with one dog achieving remission and the other experiencing relapse after initial improvement.

In six dogs, follow-up titres remained stable. Clinically, three improved without relapse, one was euthanised due to progressive disease, and two experienced relapses after initial improvement. Notably, one dog with multiple relapses exhibited increased *Neospora* titres during the second relapse compared to the initial levels.

### PCR testing from CSF

PCR results from CSF were reported as positive or negative in 51 out of 82 cases (62.2%). In the remaining 31 cases (37.8%), PCR was either not performed or results were unavailable. Of the tested cases, 27 (52.9%) were PCR-positive, while 24 (47.1%) were PCR-negative.

### MRI findings

A summary of MRI findings is provided in Table 2. Brain MRI was performed in 68 cases, spinal MRI in 23 cases, and combined brain and spinal MRI in 9 cases. Overall, 6.1% (5/82) of dogs had unremarkable MRI scans of the CNS and/or nerve roots/paraspinal muscles. Notably, 4 of these unremarkable scans were spinal MRIs, representing 17.4% of spinal MRI cases. Among brain MRIs, only one was normal, representing 1.5%. In two cases, although no intracranial lesions were detected, masticatory muscle lesions were present. Within spinal MRI cases, 26% (6/23) revealed no abnormalities in the spinal cord or paraspinal muscles. Two of these also had brain MRIs with detected lesions. Additionally, in two spinal MRI cases, no intraparenchymal or meningeal abnormalities were found, but paraspinal muscle involvement was noted.

**Table 2:** Summary of MRI Findings in 82 MRI-confirmed cases of canine CNS neosporosis.

Domain	Parameter	Finding
MRI overview	Brain MRI performed	68 cases
	Spinal MRI performed	23 cases
	Combined brain + spinal MRI	9 cases
	Normal MRI overall	6.1% (5/82) total 4 spinal MRIs (17.4% of spinal MRIs) 1 brain MRI (1.5% of brain MRIs)
	Normal spinal MRI cases	26% (6/23) spinal MRIs 2 had brain MRIs with lesions
	Normal brain MRI cases	4.4% (3/68) brain MRIs 2 had masticatory muscle lesions only
Intracranial findings	Intraparenchymal abnormalities	95.6% (65/68) brain MRIs
	Multifocal intraparenchymal lesions	51.4% (35/68) of brain MRI cases 52.4% (43/82) of total cases
	Cerebellar lesions as the sole abnormality	22.1% (15/68) of brain MRI cases 19% of total detailed cases
	Overall cerebellar lesions	72.1% (49/68) of brain MRI cases 62% of total detailed cases
	Cerebellar atrophy	57.4% (39/68 brain MRIs) 49.4% of total detailed cases
	Cerebellar atrophy as the sole abnormality	10.3% (7/68 brain MRIs) 8.9% of total detailed cases
Spinal findings	Intraparenchymal/nerve root changes	56.5% (13/23) spinal MRIs
	Concurrent brain + spinal lesions	6.3% (5/79) total detailed cases
	Spinal cord atrophy	13% (3/23) spinal MRIs
Contrast enhancement	Brain MRI with contrast data (*n* = 57)	Enhancement in 42.1% (24/57) Meningeal enhancement alone: 14% (8/57) Intraparenchymal enhancement alone: 29.8% (17/57) Both meningeal + intraparenchymal: 5.3% (3/57)
	Spinal MRI contrast (*n* = 23)	Overall enhancement in 39.1% (9/23) Intraparenchymal/nerve root enhancement in 21.7% (5/23) Meningeal enhancement only: 17.4% (4/23) Both meningeal + intraparenchymal: 8.7% (2/23)
Muscle findings	Masticatory muscle lesions (brain MRI)	17.6% (12/68)—all contrast-enhancing
	Epaxial muscle lesions (spinal MRI)	26.1% (6/23)—all contrast-enhancing
Tissue involvement	Grey vs. white matter involvement (*n* = 47)	Grey matter only: 40.4% (19/47) White matter only: 6.4% (3/47) Both grey + white matter: 29.8% (14/47) No grey/white matter involvement: 23.4% (11/47)
Cerebellar T2W/FLAIR rim sign	T2W/FLAIR hyperintense rim with T1W hypointensity	Present in 37.5% (21/56 with cerebellar imaging description available) Variable contrast enhancement

Three cases lacked detailed MRI descriptions sufficient for regional analysis and were excluded from further evaluation, leaving 79 cases with detailed MRI data.

Intracranial intraparenchymal abnormalities were observed in 65/68 (95.6%) brain MRI cases. Multifocal intraparenchymal lesions were detected in 51.4% (35/68) of brain MRI cases and 52.4% (43/82) of total cases. The cerebellum was described to be affected in 72.1% (49/68) of brain MRI cases and in 62% (49/79) of total cases with sufficient description data. Isolated cerebellar lesions were detected in 22.1% (15/68) of brain MRI cases and 19% (15/79) of total cases with sufficient description data. Cerebellar atrophy was documented in 39 cases (57.4% of brain MRI cases; 49.4% of total cases with MRI description data), with cerebellar atrophy as the sole MRI abnormality detected in 7 cases (10.3% of brain MRI cases and 8.9% of total detailed cases). Spinal intraparenchymal and/or nerve root changes were identified in 56.5% (13/23) of spinal MRI cases. Concurrent cerebral and spinal intraparenchymal lesions were present in 6.3% (5/79) of cases.

Contrast enhancement data were missing for 11 cases. Among the 57 brain MRI cases with contrast data, enhancement was observed in 42.1% (24/57), meningeal enhancement in 14% (8/57), and cerebral intraparenchymal enhancement in 29.8% (17/57). Both intraparenchymal and meningeal enhancement co-occurred in 5.3% (3/57). For spinal MRI, contrast enhancement information was available in all cases, with 39.1% (9/23) showing contrast enhancement and 21.7% (5/23) of cases with intraparenchymal/nerve root enhancement. Meningeal enhancement alone was observed in 17.4% (4/23) of spinal MRI cases, and dual enhancement was observed in 8.7% (2/23).

Spinal cord atrophy was reported in 13% (3/23) of spinal MRI cases.

Masticatory muscle lesions were identified in 17.6% (12/68) of brain MRI cases, all showing contrast enhancement. Other affected muscle groups, such as paraspinal and hind limb musculature, were reported in 26.1% (6/23) of spinal MRI cases, identified by abnormal signal intensity and contrast enhancement.

MRI descriptions were insufficient to specify the involvement of white vs. grey matter in 35/82 cases. Among the other 47 cases, 11 (23.4%) had no grey or white matter involvement, 40.4% (19) had grey matter involvement, 6.4% (3) had white matter involvement, and 29.8% (14) had both grey and white matter affected.

In 21 of 56 (37.5%) cases with detailed cerebellar imaging available, T2-weighted and FLAIR (descriptions of FLAIR images were not available in 5 cases) sequences revealed hyperintense rims with corresponding hypointensity on T1-weighted images, showing variable contrast enhancement.

### Follow-up and outcomes

Treatment and outcome data summary are provided in Table 3.

**Table 3:** Follow-up, treatment, and outcomes in 82 MRI-confirmed cases of canine CNS neosporosis.

Domain	Parameter	Finding
Follow-up duration	Cases with follow-up data	68/82
	Range	0 days – 132.4 months
	Median	3.5 months
	Mean	10.42 months
Survival time	Range	0–132.4 months
	Minimal survival time median	2.65 months
	Minimal survival time mean	13.03 months
Survival beyond follow-up/publication	Number of dogs	27 dogs survived >2 to >16 months
	Clinical outcomes	5 remission, 12 improved/stable, 5 relapsed after improvement, 2 died of unrelated causes, and 2 outcome unknown
Death/euthanasia due to neosporosis	Number	25/68 (36.8%)
	Time to euthanasia	0–30.6 months (mean: 4.05, median: 0.43)
	Notes	2 euthanised before treatment; 9 died of unrelated/suspected unrelated causes
	Survival <12 months (all causes)	21/68 (30.9%)
Corticosteroid use in death/euthanasia cases	Number	10/17 with complete treatment data (58.8%)
Full remission	Number	8/68 (11.8%)
	Relapse	2/8 relapsed
	Sustained remission	6/8 (follow-up 3–34.3 months; mean: 11.72, median: 8)
	Treatment regimens	Clindamycin ± TMPS, ± corticosteroids, ± pyrimethamine
	Corticosteroid use in remission	2 cases (one with incomplete treatment data)
Improvement without relapse	Number	23/68 (33.8%)
	Follow-up	0.25–60.8 months (mean: 9.65, median: 4)
	Notes	7 died/euthanised due to unrelated causes
Relapse	Number	18/68 (26.5%)
	Interval to relapse	0.25–26 months (mean: 10.11, median: 7.05)
	Multiple relapses	3 dogs
	Corticosteroid use	7/15 with treatment data (46.7%)
Sustained improvement or remission without relapse	Number	31/68 (45.6%)
	Treatment duration	1–11 months (mean: 4.18; median: 3.45)
	Treatment ongoing at the last follow-up	8 cases
	Common antibiotic regimens	Clindamycin + TMPS, clindamycin alone, TMPS + pyrimethamine, or other combinations
	Prednisolone use in complete treatment data cases	10/25 (40%)
Overall treatment duration (all cases)	Range	0–11 months
	Mean	2.5 months
	Median	2 months
	Missing data	28 cases
	Ongoing treatment	10 cases at the last follow-up

TMPS—Trimethoprim–sulphonamide.

Outcome data were reported in 68/82 cases, with follow-up durations ranging from 0 days to 132.4 months (median: 3.5 months; mean: 10.42 months). Twelve cases lacked time of follow-up information. Survival times ranged from 0 to 132.4 months, with a minimal survival time median of 2.65 months and a mean of 13.03 months; 13 cases had no survival data.

Twenty-seven dogs survived beyond the follow-up or manuscript publication date, with survival times exceeding 2 to over 16 months. Of these, 5 were in remission, 12 showed improvement or stable status, and 5 experienced relapses following initial improvement. Two dogs died later from unrelated or suspected unrelated causes, and clinical outcome data were unavailable for 2 dogs.

Twenty-five of 68 dogs (36.8%) were euthanised or died due to disease progression or poor quality of life associated with neosporosis, with time to euthanasia ranging from 0 to 30.6 months (mean: 4.05 months; median: 0.43 months). In two cases, euthanasia was elected prior to treatment initiation. Nine dogs died due to unrelated or suspected unrelated causes. Twenty-one cases (30.9%) survived less than 12 months post-diagnosis regardless of cause of death.

Corticosteroids were administered in 10 of the 17 cases with full treatment data who died or were euthanised due to neosporosis (58.8%). Medication details were incomplete in 8 of the 25 euthanised/died cases.

Full remission was documented in 8 of 68 dogs (11.8%), although 2 of these later relapsed at unspecified time points post-diagnosis. For the 6 dogs with remission without relapse, follow-up ranged from 3 to 34.3 months (mean: 11.72; median: 8 months). Remission was achieved under various antimicrobial treatment regimens including clindamycin and trimethoprim–sulphonamide (TMPS) combinations, with or without corticosteroids and pyrimethamine. Corticosteroids were used in two cases, going into remission without a report of relapse; however, in one of the cases, the full treatment protocol was not provided.

Improvement without relapse, meaning partial or incomplete neurological recovery maintained throughout follow-up, was observed in 23 dogs (33.8%), with follow-up from 0.25 to 60.8 months (mean: 9.65; median: 4 months). Seven of these dogs died or were euthanised for reasons unrelated to neosporosis.

Relapses occurred in 18 of 68 dogs (26.5%), with documented relapse intervals ranging from 0.25 to 26 months post-diagnosis (mean: 10.11; median: 7.05 months). Three dogs had multiple relapses. Corticosteroids were used in 7 of 15 cases with treatment data available (46.7%).

In total, 31 of 68 dogs (45.6%) showed sustained improvement or remission without relapse, deterioration, or death related to neosporosis. Treatment duration ranged from 1 to 11 months (mean: 4.18; median: 3.45 months). Treatment duration was unknown in 5 cases, and treatment was ongoing at the last follow-up/manuscript publication in 8 cases. Antibiotic regimens commonly included clindamycin and TMPS, clindamycin alone, TMPS with pyrimethamine, or various combinations. Prednisolone was included in the treatment protocol in 40% (10/25) of cases with complete treatment data.

Across all cases, treatment duration ranged from 0 to 11 months (mean: 2.5; median: 2 months). Duration was unspecified in 28 cases and ongoing in 10 cases at the last follow-up.

### Statistical analysis

**Figure 1:**
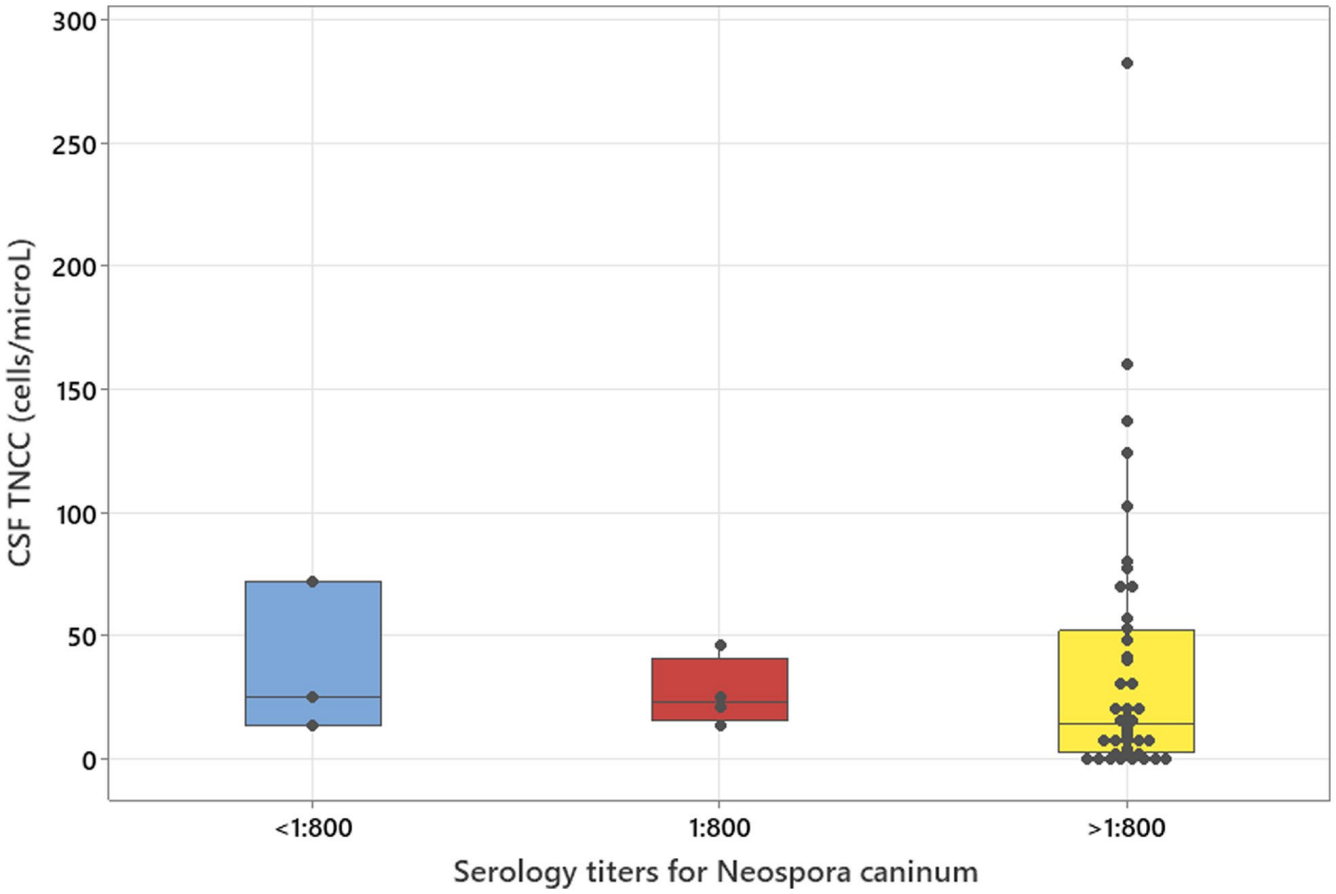
Kruskal–Wallis test adjusted for ties results to analyse three groups of serology titres for *Neospora caninum* in relation to CSF TNCC (*p* = 0.506).

**Figure 2:**
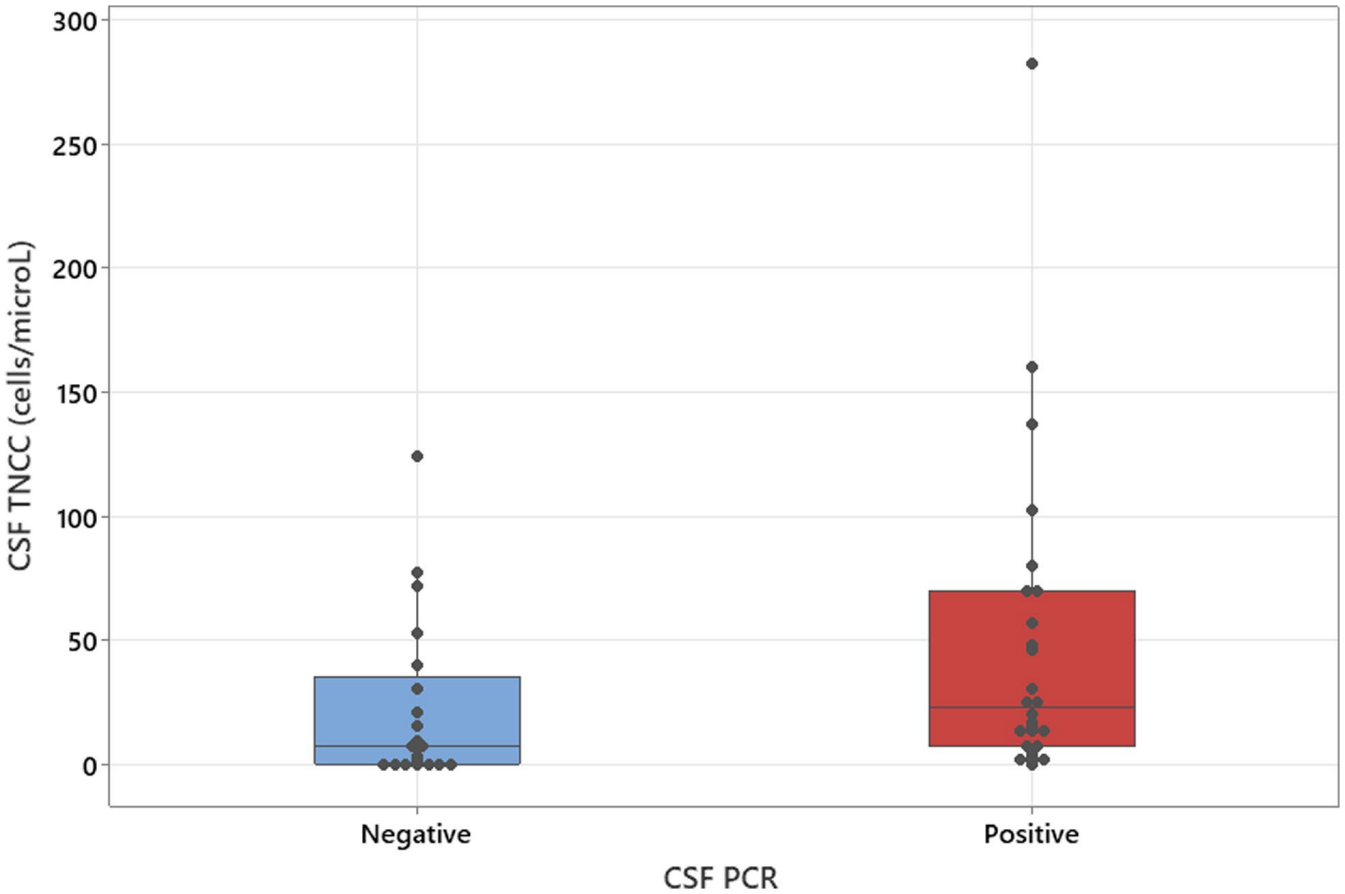
Mann–Whitney U-test adjusted for ties results to analyse CSF PCR in relation to CSF TNCC (median values: 7 and 22; *p* = 0.031).

**Figure 3:**
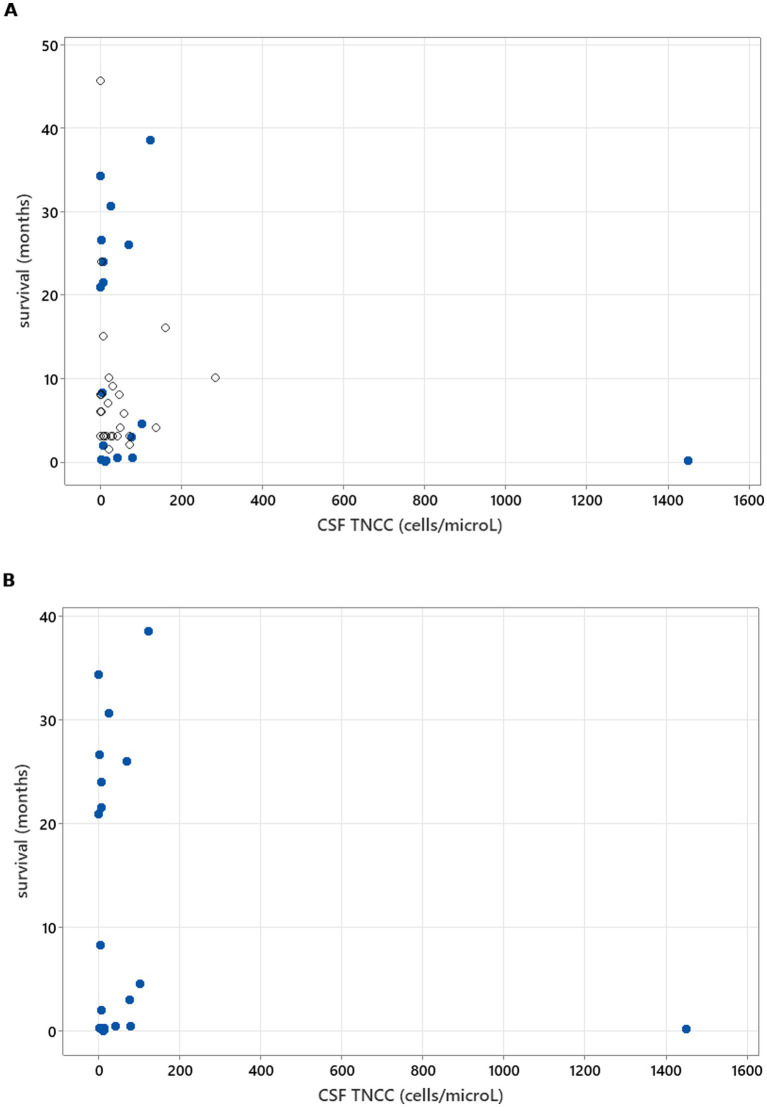
Spearman’s rank correlation test results to analyse the correlation between CSF TNCC and survival. **(A)** illustrates CSF TNCC correlation with survival in all cases (r_s_ = −0.171, *p* = 0.236). **(B)** illustrates CSF TNCC correlation with survival in patients who died/were euthanised due to neosporosis (r_s_ = −0.130, *p* = 0.563).

**Figure 4:**
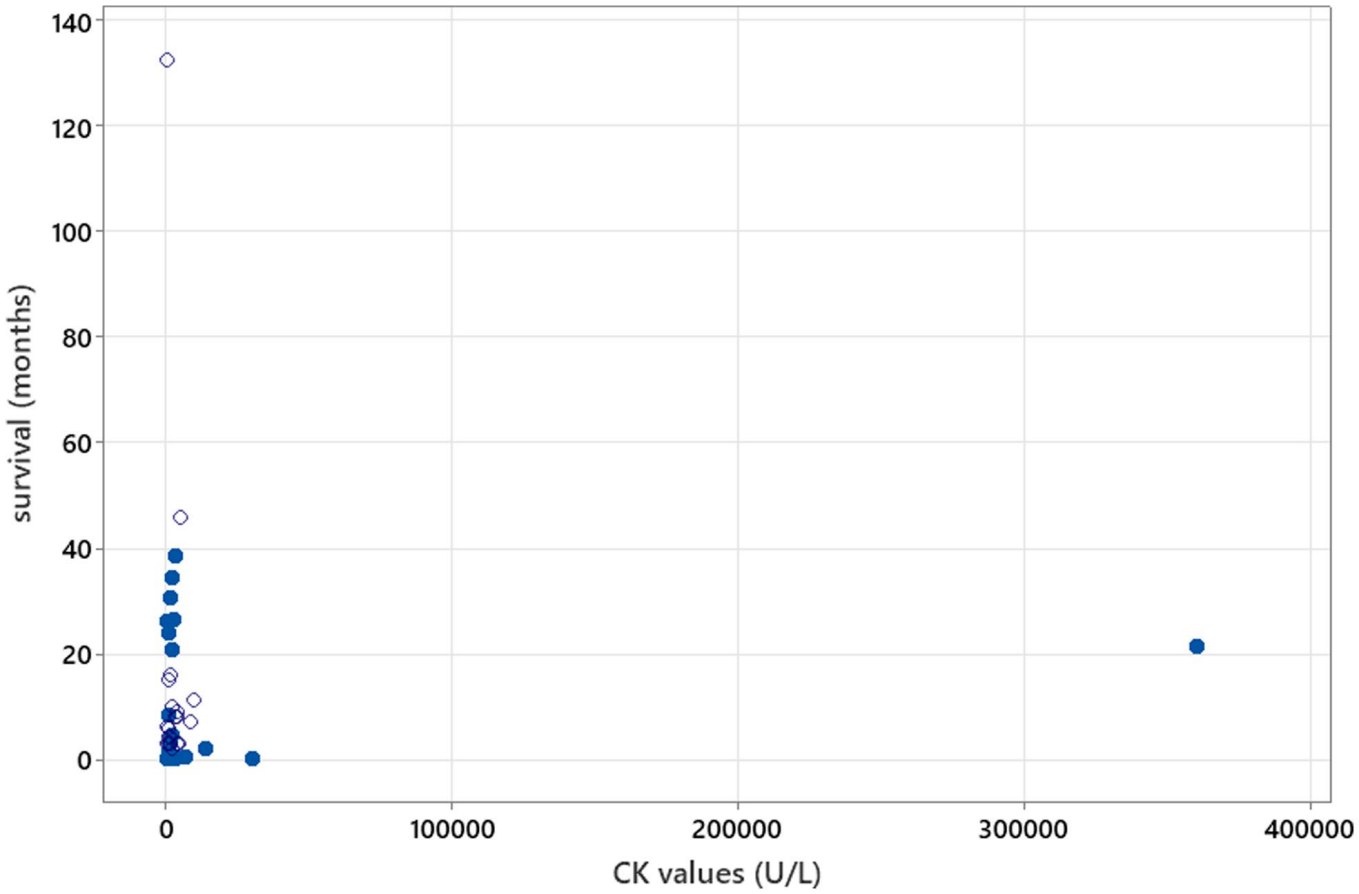
Spearman’s correlation analysis to investigate CK value correlation with survival (r_s_ = 0.044, *p* = 0.772). Open circles—patients who were still alive at the time of writing of the manuscript/time of follow-up. Blue-filled circles—deaths/euthanasia related to neosporosis.

**Figure 5:**
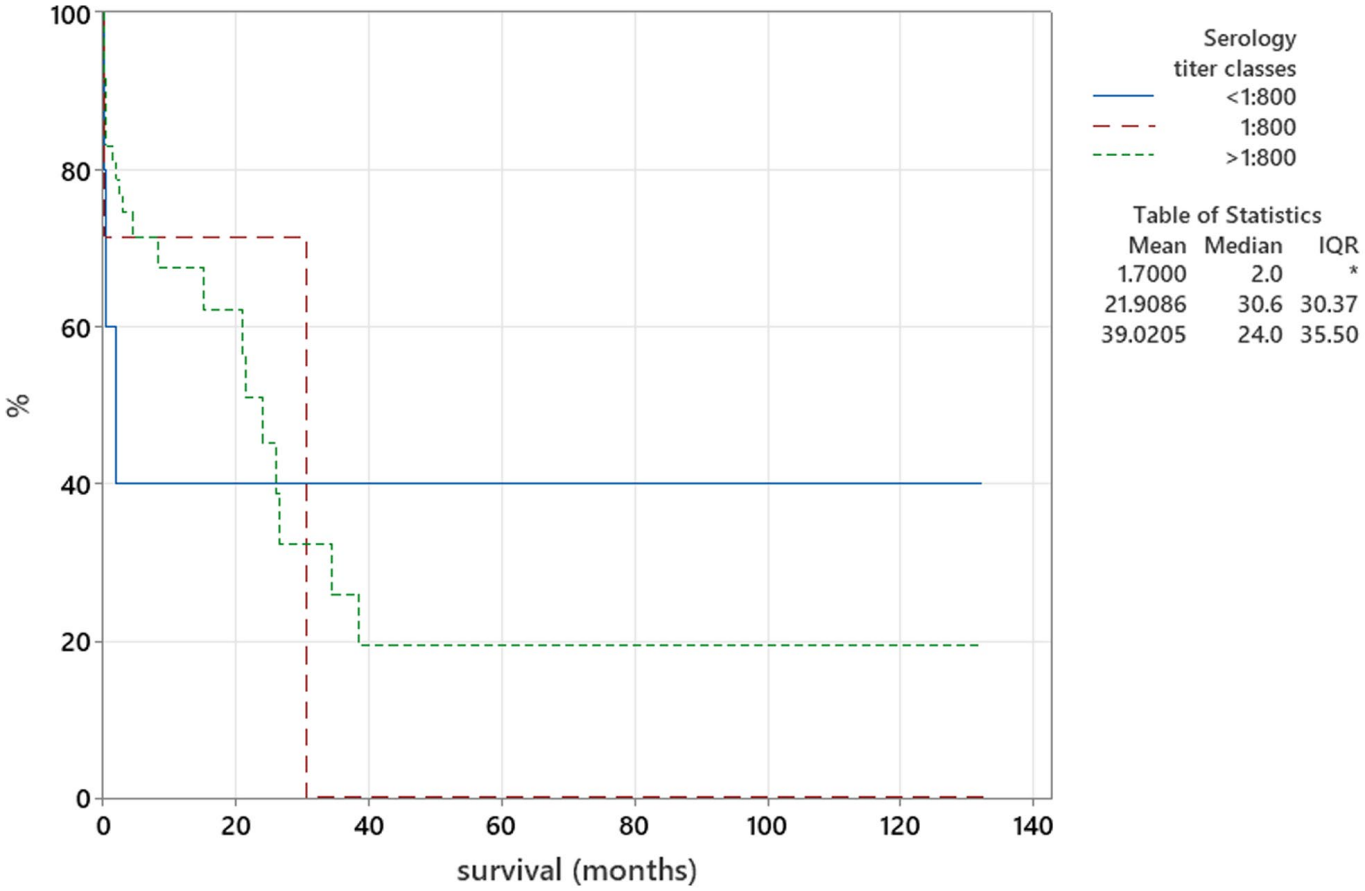
Kaplan–Meier plot tested using the log-rank test to illustrate serology titres for *Neospora caninum* in relation to survival (*p* = 0.172).

**Figure 6:**
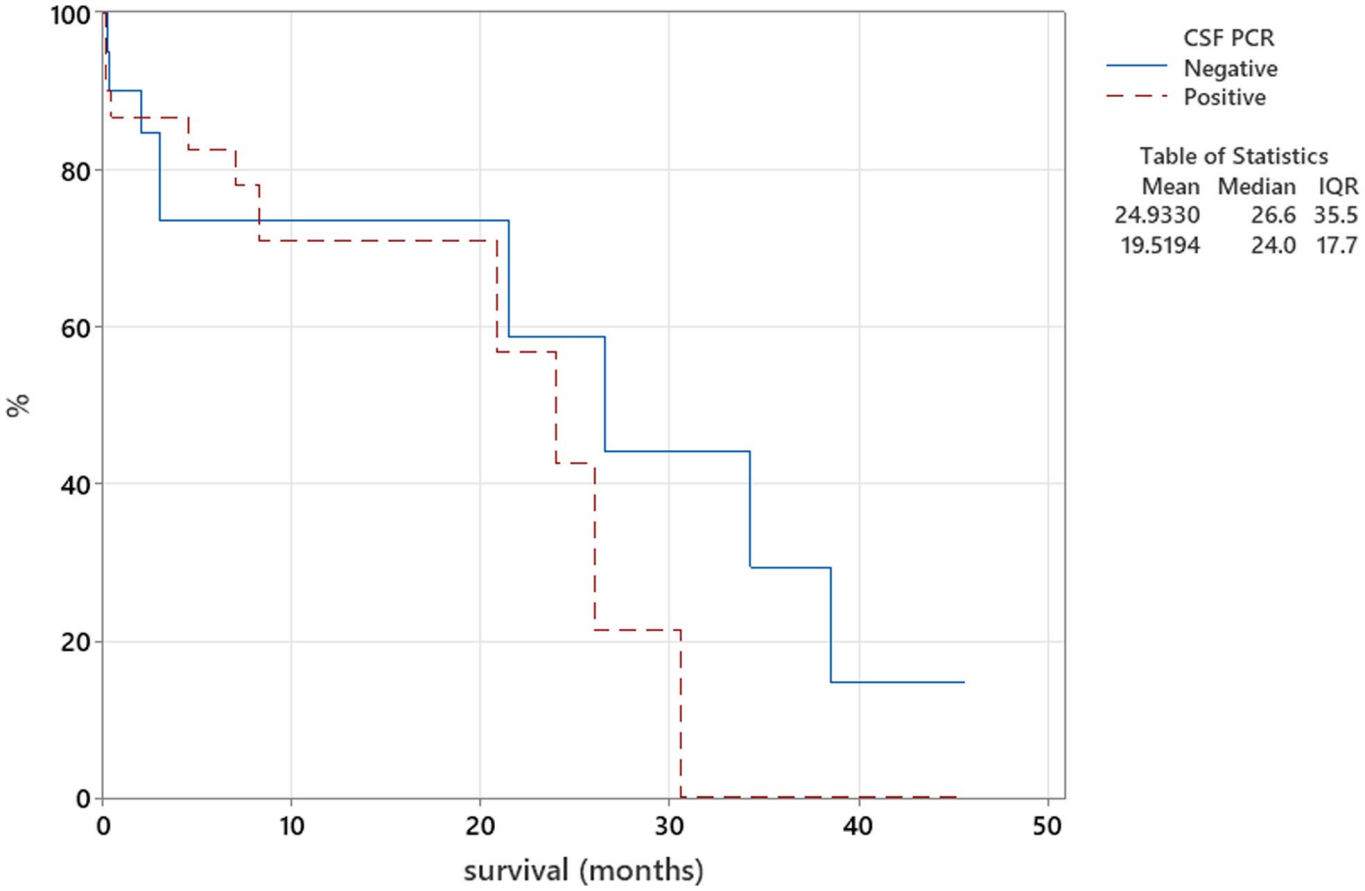
Kaplan–Meier plot tested using the log-rank test to illustrate CSF PRC for *Neospora caninum* in relation to survival (*p* = 0.559).

**Figure 7:**
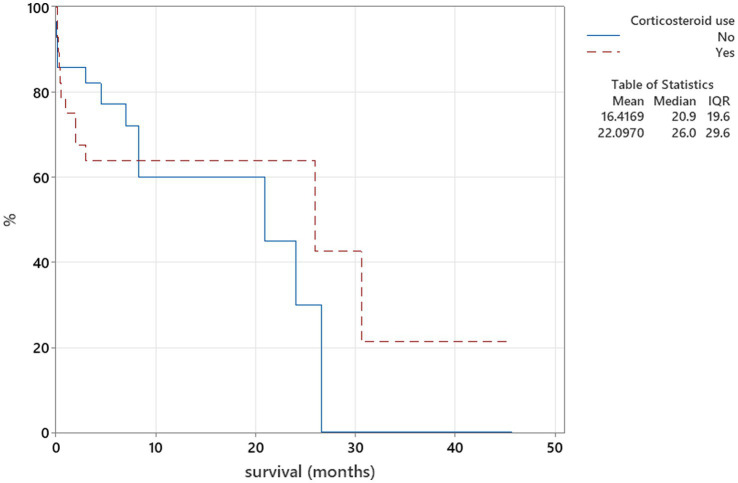
Kaplan–Meier plot tested using the log-rank test to illustrate corticosteroid use as part of the treatment protocol for neosporosis in relation to survival (*p* = 0.985).

**Figure 8:**
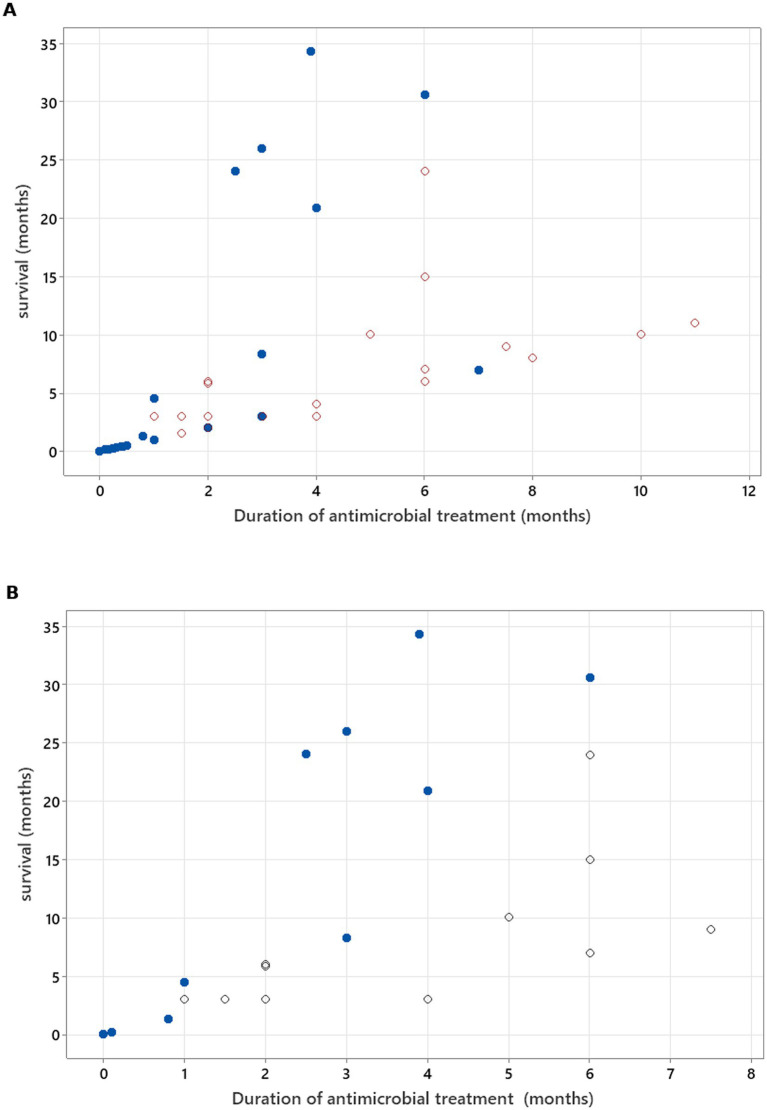
**(A)** Spearman’s correlation analysis to investigate treatment duration correlation with survival in all cases included (r_s_ = 0.868, *p* < 0.001). **(B)** Spearman’s correlation analysis to investigate treatment duration correlation with survival, excluding cases where, at the time of death/euthanasia, animals were being treated with antimicrobials (r_s_ = 0.722, *p* < 0.001). Open circles—patients who were alive at the time of writing of the manuscript/time of follow-up. Blue-filled circles—deaths/euthanasia related to neosporosis.

**Figure 9:**
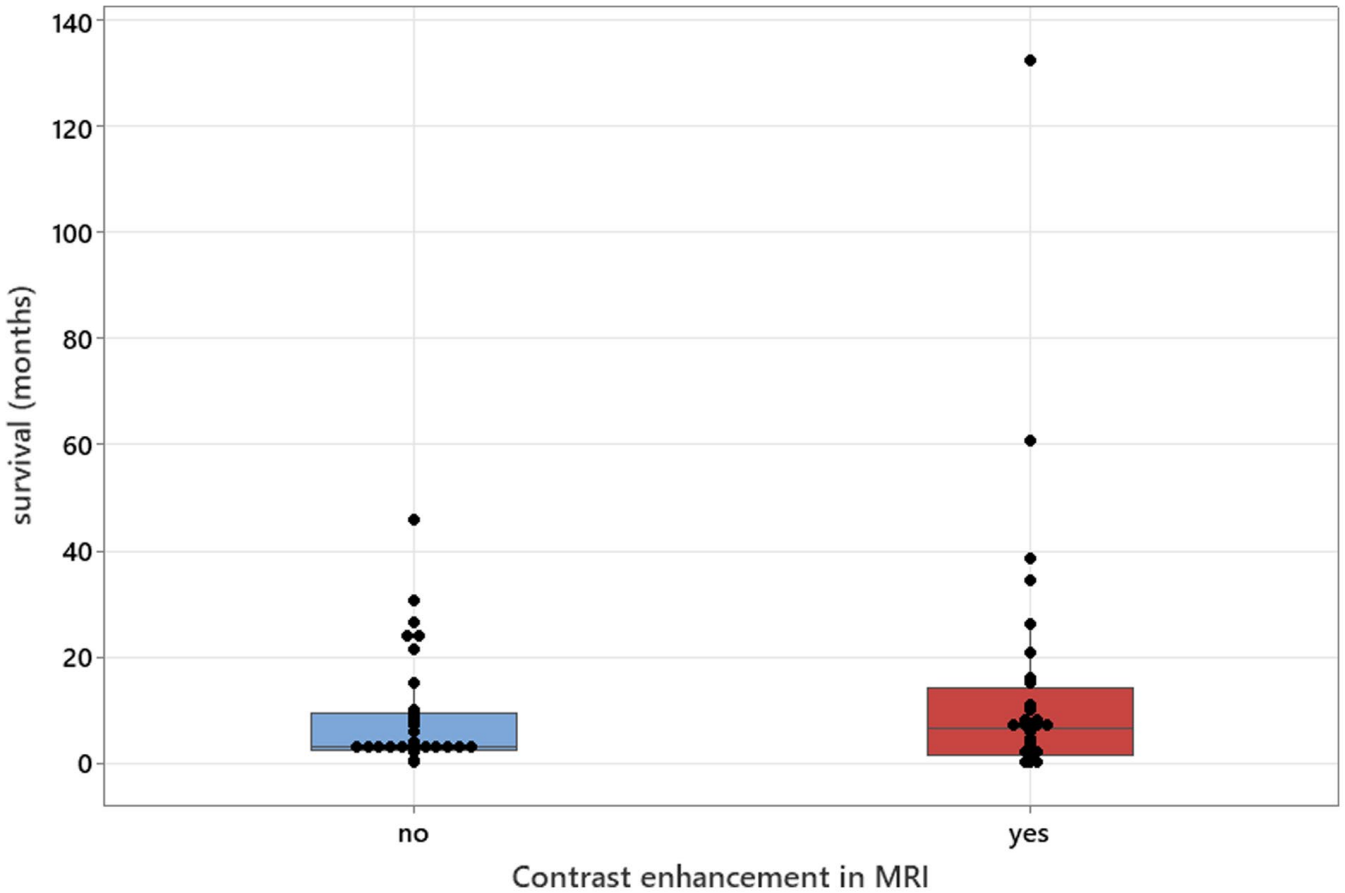
Mann–Whitney U-test adjusted for ties results to analyse contrast enhancement in MRI studies in relation to survival (median values: 34 and 32; *p* = 0.571).

There was no relation between serology titres for *Neospora caninum* and CSF TNCC found (*p* = 0.506) (Figure 1). There was a statistically significant difference observed between CSF PCR results and CSF TNCC (*p* = 0.031) (Figure 2). Cases that were CSF PCR-positive for neosporosis were more likely to have a higher CSF TNCC. Higher serology titres were not related to positive or negative PCR from CSF findings (*p* = 0.863). There was no correlation between CSF TNCC or CK values and survival found (Figures 3, 4). There was no relation between serology titres and survival identified (*p* = 0.172) (Figure 5). There was no statistically significant relation between CSF PCR test results and survival identified (*p* = 0.559) (Figure 6). There was no statistically significant relation between corticosteroid use as part of the treatment protocol for neosporosis and survival detected (*p* = 0.985) (Figure 7). There was a statistically significant correlation between the duration of treatment and survival identified (Figure 8). For all cases included in the analysis, the values were r_s_ = 0.849 and *p* < 0.001 (Figure 8, graph A); for cases excluding the ones where, at the time of death\euthanasia, animals were being treated with antimicrobials, the values were r_s_ = 0.722, p < 0.001 (Figure 8, graph B). Dogs that were treated for a longer duration were more likely to survive longer. There was no statistically significant relation between contrast enhancement described in MRI scans and the survival of the patients (Figure 9).

## Discussion

This review analysed 82 published MRI-confirmed cases of canine neosporosis with CNS involvement. Labrador Retrievers (21/82, 25.6%) and Greyhounds (18/82, 21.9%) were the most common breeds, consistent with previous studies where retrievers and sighthounds are overrepresented (3, 6–8). These findings also raise the question of potential genetic susceptibility, which warrants further investigation through breed-specific seroprevalence studies or genomic analyses. The median age at presentation was 4.5 years (range: 0.3–12 years), with the majority of dogs affected as adults (>1 year), supporting findings from previous reports (3, 6, 7). This supports the observation that CNS neosporosis is predominantly a young-to-middle-aged adult disease. Younger dogs more often develop neuromuscular forms, reported to have a median onset of 10.5 months, which are less likely to prompt CNS imaging (1, 4, 10). The majority of cases originated from the UK (65/82) and Australia (13/82), reflecting the location of contributing referral hospitals (2, 3, 5, 7, 8), and suggesting that CNS neosporosis may be underdiagnosed in regions with limited MRI access. Raising awareness of the neurological form of the disease in other countries will become increasingly important as MRI accessibility and availability expand. Encouraging testing for neosporosis is crucial—serology, MRI examination, and CSF tap with PCR are often sufficient to reach a diagnosis.

Multifocal CNS involvement was the most common localisation (57.3%), with cerebellar signs in 40% of dogs, often alongside brainstem, forebrain, or spinal cord deficits. These findings align with previous reports and emphasise the pathogen’s neurotropism and ability to disseminate widely within the CNS (3, 6, 7). Although cerebellar atrophy and necrotising cerebellitis have been historically regarded as hallmark lesions (2, 8), recent larger series show a broad spectrum of localisations beyond the cerebellum (3, 6, 7). In our review, cerebellar signs were the sole finding in only 16.9% of cases, indicating that they are neither required nor sufficient for diagnosis. As previously suggested, our findings confirm that adult dogs are more likely to suffer CNS forms of neosporosis than PNS forms; however, in contrast to historical emphasis on a purely cerebellar presentation, they more commonly exhibit multifocal CNS disease.

Limb paresis was more common than cerebellar ataxia (49.3% vs. 23.2%), highlighting that clinicians examining dogs with paresis should consider neosporosis as a differential diagnosis (3). Localisation solely within the spinal cord occurred in 23.1% of dogs, with cervical and thoracolumbar segments more commonly affected than the traditionally expected L4–S1 localisation (3). This pattern may be underrecognised and should prompt broader MRI coverage in suspected cases.

Sole peripheral nervous system involvement was found in 5.8% of cases, with an additional 11.6% showing combined CNS and PNS pathology. This highlights that PNS disease is not confined to puppies and may be overlooked in adults (4, 10). Electromyography and muscle biopsy remain valuable, particularly when MRI does not reveal clear CNS abnormalities.

CSF abnormalities were common (94.1%), with pleocytosis in 79.4%, consistent with previous reports (2, 3, 5–8). While it has been suggested that the CSF of dogs with *N. caninum* meningoencephalitis may sometimes be normal (11), review findings indicate this is uncommon, supporting CSF analysis as a valuable diagnostic tool. ACD occurred in 14.7% of cases, confirming that elevated protein with normal cell counts does not exclude infection (3, 5–8). Cytology patterns were varied, with mononuclear pleocytosis most frequent (27.9%), followed by mixed cell (23.5%), eosinophilic (14.7%), and neutrophilic (11.7%) forms, a distribution pattern that has also been noted previously (11). Eosinophilic and neutrophilic pleocytoses—although less common—should be recognised as possible indicators of neosporosis and warrant targeted diagnostic testing, including serology and CSF PCR.

Serum CK was elevated in 88.5% of dogs, aligning with prior studies (3, 11, 12), but was not associated with survival, as previously suggested (3). AST and ALT elevations were also frequent (3, 7, 11, 12). Although ALT has been described as only occasionally increased in neosporosis (11), review data indicate that it may be an important marker, with 79.3% (23/29) of reported cases showing elevated values. This likely reflects hepatic or extrahepatic (particularly muscle) involvement, supporting the inclusion of ALT measurement in the standard biochemical workup for suspected CNS neosporosis.

High serology titres (≥1:800) were present in 93.6% of cases, supporting their diagnostic utility (3, 6, 7, 11, 12), although lower titres do not exclude disease, particularly in neuromuscular cases (1, 4). Combining serology with CSF PCR and follow-up testing improves accuracy. Titre trends may reflect disease course but require clinical context. CSF PCR was positive in 52.9% of tested dogs, similar to other large studies (3, 6), while other authors reported variable CSF PCR results (4, 5, 7). This supports our review finding that while certain laboratory values, such as higher CSF TNCC, may coincide with positive PCR results, other expected associations—such as between serology titres and PCR status—were not statistically significant in our analysis. Sensitivity appears influenced by disease stage and CSF cellularity, and negative results do not exclude infection. Muscle biopsy or repeat sampling can improve detection in selected cases.

MRI abnormalities were detected in 98.5% of brain studies and in 74% of spinal studies, confirming higher diagnostic yield from brain imaging (3, 7). Multifocal intracranial lesions were detected in over half of the cases, with a similar prevalence of cerebellar atrophy, which was observed alongside other lesions (3, 5, 7). Contrast enhancement was present in 42.1% of brain MRIs and 39.1% of spinal MRIs, but its absence does not exclude disease and, contrary to prior assumptions, was not significantly associated with longer survival in our dataset (3).

A distinctive cerebellar rim pattern—T2W/FLAIR hyperintensity with peripheral enhancement—was identified or suspected in over one-third of evaluable cases, consistent with previous descriptions (2–4, 8). While this sign may aid diagnosis, its absence does not preclude pathology, as observed in cases undergoing post-mortem examinations (2, 13).

Extracranial muscle involvement was frequent, affecting paraspinal (26.1%) and masticatory (17.6%) muscles, and when present, was always associated with contrast enhancement. Such lesions may be the only abnormality when CNS imaging appears normal, emphasising the importance of including muscle evaluation in MRI protocols.

Grey matter was affected in 70.2% of evaluable cases, supporting the parasite’s neurotropism, which was observed previously (3, 8, 10, 13, 14). Unremarkable MRIs occurred in 6.1% of dogs, mostly in spinal studies (17.4% of spinal MRIs vs. 1.5% of brain MRIs), and sometimes muscle lesions were the sole abnormal finding.

While earlier textbook descriptions summarised typical cerebellar and meningeal changes in MRI (11), our review offers a much broader perspective, capturing lesion distribution patterns across the CNS and PNS, the consistent presence of extracranial muscle enhancement, and the prevalence of the cerebellar T2W/FLAIR rim sign.

Overall, MRI in neosporosis reveals predominantly multifocal, asymmetric, grey matter–predominant lesions with variable contrast enhancement. Cerebellar involvement is common, and the cerebellar T2W/FLAIR rim sign is a useful marker but not universally present. In contrast to brain MRI, normal spinal imaging should not exclude neosporosis, and systematic assessment of paraspinal and masticatory muscles—especially with contrast—can improve diagnostic sensitivity. Conversely, an unremarkable MRI in dogs with intracranial signs is unlikely to be associated with neosporosis, highlighting the need for judicious interpretation when considering differential diagnoses.

Follow-up data showed neurological improvement or remission without documented relapse in 45.6%, but full and sustained remission was rare (8.8%), and relapse occurred in 26.5%—most within the first year. Mortality due to neosporosis was high (36.8%), aligning with reports that CNS neosporosis carries a guarded prognosis (3, 4, 7, 11). It has been emphasised that early recognition and initiation of appropriate antiprotozoal therapy are critical to improving outcomes, as delays can allow irreversible CNS damage (11), which may be true but still unknown up to this time point.

Clindamycin, alone or combined with TMPS, was the most common regimen among dogs achieving remission or sustained improvement, supporting its role as first-line therapy (3, 4, 11). Literature recommendations suggest that clindamycin at appropriate CNS-penetrating doses, with or without TMPS, should be continued for at least 8 weeks as a minimum course, with extension to an undefined period of time provided clinical improvement is still observed (11). In our review, antimicrobial durations for successfully treated cases ranged from 1 to 11 months (median 3.45 months), and longer therapy correlated significantly with improved survival, supporting these extended treatment recommendations.

Corticosteroid use was common but inconsistently associated with outcome across studies (3, 4, 7). While corticosteroids may be indicated to control severe inflammation or raised intracranial pressure, caution has been advised because immunosuppression can exacerbate protozoal replication and worsen prognosis (3, 4, 11). In our statistical analysis, corticosteroid use was not significantly linked to outcome, though variability in dosing, timing, and duration limits interpretation. It has been suggested that corticosteroids, if used, should be reserved for cases with clear evidence of detrimental inflammatory sequelae, given at the lower anti-inflammatory dose, and tapered as rapidly as clinically possible (11). Our review findings support this notion; however, based on information currently available, one could not assess adherence to such protocols, highlighting the need for future prospective studies to evaluate the safety and efficacy of corticosteroids in canine CNS neosporosis.

No significant associations were found between survival and CSF TNCC, CSF PCR status, CK values, or serology titres (Figures 3–6), suggesting that these markers have limited prognostic value and should not deter treatment initiation. This aligns with the position that laboratory results should be interpreted in a clinical context, and treatment should proceed once a diagnosis is established or strongly suspected, rather than delayed pending perfect diagnostic certainty (11).

Overall, remissions are rare, clinical improvement is achievable in many dogs with CNS neosporosis, but relapse, deterioration, and death are frequent. Combining early diagnosis, prolonged antimicrobial therapy, judicious corticosteroid use, and structured follow-up, especially in the first year after diagnosis, offers the best chance of improving long-term outcomes. Prospective, standardised studies are needed to define optimal treatment protocols, clarify the impact of corticosteroids, and identify reliable prognostic indicators.

## Limitations

This study is a retrospective review of published cases, introducing variability in clinical documentation, diagnostic criteria, MRI protocols, and treatment approaches. The inconsistencies with MRI sequence availability and descriptions limited the detection of subtle features such as the cerebellar FLAIR rim sign or specific grey/white matter patterns. Due to inter-laboratory variations, bloodwork, serology, and PCR tests had varying cutoff thresholds (mostly for serology), which made direct comparisons difficult. Treatment regimens for antimicrobial agents and corticosteroids were inconsistently documented, and follow-up was often incomplete or variably defined. Selection bias is likely, as only MRI-confirmed cases with definitive diagnoses were included, potentially overrepresenting more severe or atypical presentations.

## Conclusion

This review provides the most comprehensive synthesis to date of MRI-investigated CNS neosporosis in dogs. The disease predominantly affects young-to-middle-aged adults, with certain breeds overrepresented, and usually presents with multifocal CNS involvement rather than isolated cerebellar disease. Cervical and thoracolumbar spinal localisations are common, and paraparesis or tetraparesis should prompt consideration of neosporosis.

CSF is abnormal in the majority of cases, most often showing mononuclear or mixed cell pleocytosis. Serum ALT is frequently elevated, alongside previously investigated CK and AST. High serology titres (≥1:800) strongly support diagnosis, though lower titres do not exclude the disease. MRI findings extend beyond early descriptions, with grey matter–predominant lesions, the cerebellar T2w/FLAIR rim sign, and extracranial muscle lesions and contrast enhancement supporting inclusion of adjacent musculature in imaging protocols. Cerebellar involvement was commonly detected, but rarely in isolation, as lesions were most often found in combination with other CNS abnormalities.

Analysis showed that serology titres, serum CK values, and MRI contrast enhancement had no prognostic value. Neither TNCC nor CSF PCR results correlated with outcomes, although higher TNCC was significantly associated with positive PCR detection. The only consistent prognostic marker was the duration of antimicrobial treatment, with longer courses strongly improving survival. Corticosteroid use showed no clear survival effect but should be approached cautiously.

Overall, prognosis remains guarded, but early recognition and prolonged antimicrobial therapy—typically clindamycin ± TMPS, with cidal agents considered when available—are key to improving outcomes (see Table 4).

**Table 4:** Practical recommendations for clinicians managing canine CNS neosporosis.

Domain	Recommendations
Awareness and surveillance	Raise awareness of the neurological form internationally, especially where MRI availability is expanding. Support surveillance and reporting efforts, as prevalence data outside the UK remain limited.
Diagnosis	Test routinely for neosporosis in dogs with compatible neurological signs (serology, MRI, CSF analysis, and PCR). Consider neosporosis in young-to-middle-aged dogs with multifocal CNS signs or paresis, including cervical or thoracolumbar localisation.
CSF and biochemistry	Expect CSF abnormalities in the majority of cases—commonly mononuclear or mixed pleocytosis, though eosinophilic or neutrophilic forms may occur. Elevated serum ALT (alongside CK and AST) is frequent and should be included in the biochemical panel.
Serology	High titres (≥1:800) strongly support diagnosis, but lower titres do not exclude disease. Follow-up titres may reflect disease course, but should not solely guide treatment decisions.
MRI	Perform both brain and spinal MRI when indicated, and always include adjacent musculature with contrast. Cerebellar lesions are common but rarely isolated; a normal brain MRI in intracranial presentations makes neosporosis unlikely, while a normal spinal MRI does not exclude the disease.
Treatment	Use static agents (clindamycin ± TMPS) for prolonged courses—minimum 12 weeks or until clinical improvement plateaus, and at least 4 weeks beyond. Consider cidal agents (e.g., ponazuril and toltrazuril) when available. Do not rely solely on serology to determine treatment cessation.
Corticosteroids	If used, give at anti-inflammatory doses and for a short duration early in the disease; taper as soon as possible. No significant survival association was found, but immunosuppression remains a concern.
Prognosis and client communication	Prognosis remains guarded. Neither serology, CK, MRI contrast, CSF TNCC, nor PCR status predicts outcome. The only consistent prognostic marker was longer antimicrobial treatment duration, which correlated with improved survival. Owners should be counselled about relapse risk and the need for extended therapy.

UK, United Kingdom, CSF, cerebrospinal fluid, PCR, polymerase chain reaction, CNS, central nervous system, ALT, alanine aminotransferase, CK, creatine kinase, AST, aspartate aminotransferase, TNCC, total nucleated cell count.

## Data Availability

The original contributions presented in the study are included in the article/Supplementary material, further inquiries can be directed to the corresponding author.
