# Supplementary material for: A systematic review of clinical and diagnostic findings, treatment, and outcomes in canine neosporosis cases evaluated by magnetic resonance imaging
Source: Front Vet Sci. 2025 Oct 22;12:1669529. doi: 10.3389/fvets.2025.1669529 (PMC12587458; doi:10.3389/fvets.2025.1669529)
Supplement: Supplementary file 1 [file Table_1.docx]

**Supplementary data**

**Table S1. Signalment, history, physical and neurological findings**

| **Nr** | **Author, Year of Publication** | **Country of Origin** | **Breed (Neutering Status)** | **Age of Onset (Years)** | **Duration of Clinical Signs** | **Main Complaint at Presentation** | **Physical Examination Findings** | **Neurological Examination Findings** | **Neurological Localization** |
| --- | --- | --- | --- | --- | --- | --- | --- | --- | --- |
| 1 | Gaitero et al., 2006 | Spain | Labrador Retriever (FE) | 4 | 5 days, progressive | Generalized ataxia, depressed mental status  Anorexia, lethargy, hyperthermia | Unremarkable  Ophthalmic examination: bilaterally swollen optic disks | Non-ambulatory tetraparesis, stupor, intermittent decerebrate rigidity, anisocoria, absent menace bilaterally, absent PLRs, decreased right sided facial sensation, absent oculocephalic reflex  Epileptic seizure | Diffuse/multifocal (forebrain and brainstem) |
| 2 | Garosi et al., 2010 | UK | West Highland White Terrier (MN) | 5 | 3 months, progressive | Ataxia | Unremarkable | Ataxia affecting all limbs, intention tremor, proprioceptive deficits in pelvic limbs, exaggerated hopping in all four limbs, decreased oculocephalic reflex, decreased nasal sensation on the right side. | Multifocal (cerebellum and brainstem) |
| 3 | Garosi et al., 2010 | UK | West Highland White Terrier (FN) | 9 | 12 months, progressive | Ataxia, head tilt | Unremarkable | Right-sided head tilt and moderate ataxia affecting all 4 limbs. Postural reactions were abnormal on the left side. | Cerebellum |
| 4 | Garosi et al., 2010 | UK | Greyhound (FN) | 7 | 2 months, progressive with rapid deterioration | Ataxia | Unremarkable | Cerebellar ataxia, hypermetria affecting all four limbs. Postural reactions were slightly impaired in the right thoracic and pelvic limbs, and mild neck discomfort was detected on flexion. Absent menace response in the left eye. | Multifocal (cerebellum and brainstem) |
| 5 | Garosi et al., 2010 | UK | Dachshund (MN) | 10 | 5 weeks, progressive | Ataxia of pelvic limbs which progressed to generalised ataxia | Unremarkable | Depressed mental status, tetraparesis and head tremor. Postural reactions were decreased in all four limbs. Patellar reflexes were decreased bilaterally and the withdrawal reflex was weak in the left pelvic limb; the perineal reflex also was decreased on the left side. Anisocoria, decreased PLR in the left eye. Facial sensation decreased on the right, bilateral temporal muscle atrophy. Spinal hyperesthesia. | Multifocal (cerebellum, brainstem, and L4-S3 spinal cord) |
| 6 | Garosi et al., 2010 | UK | Labrador Retriever (FN) | 4 | 6 months, progressive | Generalised ataxia | Unremarkable | Marked generalised cerebellar ataxia. Conscious proprioception was abnormal in both pelvic limbs and in the right thoracic limb; hopping was abnormal in all four limbs. | Multifocal (cerebellum and brainstem) |
| 7 | Garosi et al., 2010 | UK | Labrador Retriever (MN) | 9 | 6 weeks, progressive | Pelvic limb ataxia progressed to thoracic limbs, episodes of staring vacantly | Unremarkable | Marked generalised cerebellar ataxia. Conscious proprioception was slightly impaired in the thoracic limbs and markedly impaired in the pelvic limbs; hopping responses were abnormal in all four limbs. Palpebral reflexes and menace responses were impaired bilaterally, and jaw strength was decreased. Atrophy of the masticatory muscles and bilateral facial paresis. | Multifocal (cerebellum and brainstem) |
| 8 | Garosi et al., 2010 | UK | Labrador Retriever (FN) | 1.5 | 7 months, progressive | Pelvic limb ataxia, jerky pelvic limb gait | Unremarkable | Marked truncal and limb ataxia, with a jerky pelvic limb gait. Postural responses were impaired in the pelvic limbs, head and ocular tremors | Multifocal (cerebellum and brainstem) |
| 9 | Perzefall et al., 2014 | UK | Greyhound (FN) | 9 | 2 months, progressive | Tetraparesis, intermittent knuckling of right thoracic limb | Unremarkable | Obtundation, tetraparesis, generalized ataxia, proprioceptive deficits in all four limbs, leaning and falling forwards and to the right side, decreased lateral visual field, and positional ventromedial strabismus in the right eye. | Multifocal brain (with left prosencephalic component) |
| 10 | Perzefall et al., 2014 | UK | Labrador Retriever (MN) | 2 | 1.5 years, progressive | Compulsive circling to the right | Unremarkable | Compulsive circling to the right side, a subtle right- sided head tilt, and mildly hypermetric gait in the thoracic limbs (cerebellar ataxia) | Multifocal (forebrain and cerebellum) |
| 11 | Perzefall et al., 2014 | UK | Labrador Retriever (ME) | 0.4 | 2 weeks, rapid progression | Pelvic limb ataxia, progressed to non-ambulatory tetraparesis, head tremors, incontinence | Unremarkable | Thoracic limb paresis, pelvic limb plegia – non-ambulatory, decreased/absent proprioception in all four limbs, Patellar reflexes were absent and flexor- withdrawal reflexes were reduced in both pelvic and the left thoracic limb. Reduced muscle tone was evident in both pelvic and increased muscle tone in the left thoracic limb. Mild intention tremors. | Multifocal (C1-T2, L4-S3 spinal cord segments and cerebellum) |
| 12 | Perzefall et al., 2014 | UK | Cavalier King Charles Spaniel (MN) | 3 | Several weeks, progressive | Lethargy, intermittent head tilt | A systolic heart murmur consistent with previously diagnosed mitral valve disease | Proprioceptive deficits in right thoracic and right pelvic limbs, an intermittent right sided head tilt, decreased menace responses bilaterally and mild pain on cervical and lumbar spinal palpation. | Multifocal CNS disease |
| 13 | Coelho et al., 2018 | UK | Labrador Retriever (FN) | 1 | NA | NA | NA | NA | Multifocal |
| 14 | Coelho et al., 2018 | UK | Labrador Retriever (MN) | 2 | NA | NA | NA | NA | Cerebellum |
| 15 | Coelho et al., 2018 | UK | Labrador Retriever (FE) | 0.9 | NA | NA | NA | NA | Multifocal |
| 16 | Coelho et al., 2018 | UK | Greyhound (FN) | 7 | NA | NA | NA | NA | Multifocal |
| 17 | Coelho et al., 2018 | UK | Border Collie (ME) | 7 | NA | NA | NA | NA | Multifocal |
| 18 | Coelho et al., 2018 | UK | Whippet (MN) | 2 | NA | NA | NA | NA | Multifocal |
| 19 | Coelho et al., 2018 | UK | Greyhound (FN) | 4 | NA | NA | NA | NA | Cerebellum |
| 20 | Coelho et al., 2018 | UK | West Highland White Terrier (MN) | 6 | NA | NA | NA | NA | Forebrain |
| 21 | Coelho et al., 2018 | UK | Labrador Retriever (FN) | 9 | NA | NA | NA | NA | Multifocal |
| 22 | Didiano et al., 2020 | UK | Labrador Retriever (FN) | 11 | 3 weeks,  progressive | Cerebellar ataxia | Unremarkable | Cerebellar ataxia | Cerebellum |
| 23 | Didiano et al., 2020 | UK | Saluki (MN) | 2 | 2 weeks, progressive | Pelvic limb ataxia | Unremarkable | Ambulatory paraparesis, severe pelvic limb ataxia | T3–L3 spinal cord segment |
| 24 | Didiano et al., 2020 | UK | Lurcher (FN) | 10 | 4 weeks, progressive | Pelvic limb ataxia progressed to ambulatory tetraparesis | Unremarkable | Ambulatory tetraparesis, reduced proprioception in all limbs | C1–C5 spinal cord segment |
| 25 | Didiano et al., 2020 | UK | English Springer Spaniel Cross (FE) | 1.5 | 4 weeks, progressive | Cerebellar ataxia, behavioral changes (urinating and passing faeces at home) | Unremarkable | Cerebellar ataxia | Multifocal (cerebellum and forebrain) |
| 26 | Didiano et al., 2020 | UK | Labrador Retriever (MN) | 2 | 2 weeks, progressive | Cerebellar ataxia | Unremarkable | Cerebellar ataxia - hypermetria, pronounced in pelvic limbs | Cerebellum |
| 27 | Didiano et al., 2020 | UK | Greyhound (MN) | 4 | 3 weeks, progressive | Ambulatory tetraparesis | Unremarkable | Ambulatory tetraparesis, generalized mixed cerebellar and vestibular ataxia, hypermetria in all four limbs, postural reaction deficits in all four limbs | Multifocal intracranial (cerebellum and brainstem) |
| 28 | Clark et al., 2022 | UK | Labrador Retriever (ME) | 0.4 | 1 month | Started with right pelvic limb paresis, progressed to paraparesis, tail flaccidity, urinary incontinence | Moderate bilateral generalised muscle wasting of the pelvic limbs and gluteal muscle groups | Severe bilateral plantigrade stance, plegic tail, ambulatory paraparesis, bunny hopping gait in pelvic limbs, poor pelvic limb tone, reduced withdrawal reflexes in hind limbs, patellar reflex was absent in the right pelvic limb and reduced in the left pelvic limb. Perineal reflex was absent bilaterally. | L4–Cd spinal cord segments |
| 29 | Clark et al., 2022 | UK | Greyhound (FN) | 6 | 6 weeks | Muscle wasting over the lumbosacral region, progressing to non-ambulatory paraparesis | Tacky oral mucus membranes, moderate skin tenting, tachycardia (140 beats per minute), panting and pyrexia (39.8 C ). | Non-ambulatory paraparetic, short-strided pelvic limb gait when supported. The paw placing and hop- ping responses were delayed in the left pelvic limb and absent in the right pelvic limb. The withdrawal reflexes were decreased in both pelvic limbs. There was pseudohyperreflexia of the right patellar reflex, which appeared to be increased due to the decrease in tone in the antagonistic muscle groups, but despite this, there was reduced quadriceps muscle tone, suggestive of reduced femoral nerve function. The cutaneus trunci reflex was absent. | L4–S1 spinal cord segments, nerve roots or spinal nerves |
| 30 | Alf et al., 2024 | unknown | English Springer Spaniel (F) | 8 | 4 weeks | NA | NA | NA | Multifocal: CNS and PNS |
| 31 | Alf et al., 2024 | unknown | Crossbreed (F) | 2 | 3 weeks | NA | NA | NA | Multifocal: CNS and PNS |
| 32 | Alf et al., 2024 | unknown | Cane Corso (M) | 7 | 6 months | NA | NA | NA | Multifocal: CNS and PNS |
| 33 | Fisher et al., 2024 | UK | Lurcher (FN) | 7 | NA | NA | NA | Back pain, urinary incontinence | T3-S1 spinal cord segments |
| 34 | Fisher et al., 2024 | UK | Tibetan Terrier (MN) | 9 | NA | NA | NA | Ambulatory paraparesis | T3-L3 spinal cord segments |
| 35 | Fisher et al., 2024 | UK | Alaskan Malamute (MN) | 2 | NA | NA | NA | Seizures. Ambulatory, mild proprioceptive deficits pelvic limbs (suspected anti-epileptic drug related) | Forebrain |
| 36 | Fisher et al., 2024 | UK | Lurcher (MN) | 7 | NA | NA | NA | Ambulatory generalised ataxia, postural reaction deficits (right side), seizures | Multifocal (forebrain and cerebellum) |
| 37 | Fisher et al., 2024 | UK | Lurcher (ME) | 1.8 | NA | NA | NA | Ambulatory paraparesis, crossed-extensor reflex pelvic limbs, cutaneous trunci reflex cut-off T13-L1 bilaterally | T3-L3 spinal cord segments |
| 38 | Fisher et al., 2024 | UK | Golden Retriever (MN) | 3 | NA | NA | NA | Ambulatory generalised ataxia, tetraparesis, right-sided head tilt, absent menace OD | Multifocal (forebrain and brainstem) |
| 39 | Fisher et al., 2024 | UK | WHWT (MN) | 2 | NA | NA | NA | Ambulatory paraparetic, reduced withdrawals pelvic limbs, stiff stilted pelvic limb gait | Multifocal (T3-L3 spinal cord segments and L6-S1 spinal cord segments /sciatic neuropathy) |
| 40 | Fisher et al., 2024 | UK | Lurcher (FN) | 10 | NA | NA | NA | Ambulatory tetraparetic and neck pain | C1-C5 spinal cord segments |
| 41 | Fisher et al., 2024 | UK | Whippet (FE) | 0.3 | NA | NA | NA | Non-ambulatory paraparetic, cutaneous trunci reflex cut-off at L1 bilaterally | T3-L3 spinal cord segments |
| 42 | Fisher et al., 2024 | UK | Border Collie (FN) | 12 | NA | NA | NA | Ambulatory, circling (to the left), right-sided head tilt, proprioceptive deficits all four limbs, reduced menace OD, positional nystagmus | Multifocal (forebrain and brainstem) |
| 43 | Fisher et al., 2024 | UK | Cocker Spaniel (MN) | 11 | NA | NA | NA | Ambulatory tetraparetic, generalised ataxia, generalised tremors, right-sided head tilt, absent menace OD | Multifocal (forebrain and cerebellum) |
| 44 | Fisher et al., 2024 | UK | Greyhound (MN) | 3 | NA | NA | NA | Ambulatory generalised ataxia, truncal sway, absent menace OU | Cerebellum |
| 45 | Fisher et al., 2024 | UK | Greyhound (MN) | 4 | NA | NA | NA | Ambulatory generalised ataxia, generalised tremors, truncal sway | Cerebellum |
| 46 | Fisher et al., 2024 | UK | Boxer (ME) | 5 | NA | NA | NA | Ambulatory, proprioceptive deficits all four limbs, obtundation, generalised muscle wastage, right-sided head tilt, abnormal oculocephalic reflex OU, spontaneous nystagmus | Multifocal (forebrain, brainstem, myopathy) |
| 47 | Fisher et al., 2024 | UK | Labrador Retriever (FE) | 0.4 | NA | NA | NA | Ambulatory paraparesis, caudal thoracic cutaneous trunci cut-off bilaterally | T3-L3 spinal cord segments |
| 48 | Fisher et al., 2024 | UK | Greyhound (MN) | 6 | NA | NA | NA | Ambulatory generalised ataxia, tetraparesis, head tilt (to the right side) | C1-C5 spinal cord segments |
| 49 | Fisher et al., 2024 | UK | Greyhound (MN) | 6 | NA | NA | NA | Non-ambulatory, obtundation, postural reaction deficits (to the right side), reduced withdrawals all four limbs, absent menace OS, spontaneous nystagmus | Multifocal (forebrain, cerebellum and neuromuscular) |
| 50 | Fisher et al., 2024 | UK | Crossbreed (MN) | 5 | NA | NA | NA | Ambulatory generalised ataxia, generalised tremor, right-sided head tilt, delayed proprioceptive placement left pelvic limb, spontaneous vertical nystagmus, absent menace OD, intermittent menace OS | Multifocal (cerebellum and T3-L3 spinal cord segments) |
| 51 | Fisher et al., 2024 | UK | French Bulldog (MN) | 8 | NA | NA | NA | Ambulatory, proprioceptive deficits all four limbs, obtundation, facial paresis (left side), positional nystagmus, myoclonus | Multifocal (forebrain and brainstem) |
| 52 | Fisher et al., 2024 | UK | Labrador Retriever (ME) | 0.4 | NA | NA | NA | Ambulatory generalised ataxia, left-sided head tilt, ventromedial strabismus OS, absent menace OS, hemiparetic (left side), reduced withdrawal left forelimb, absent cutaneous trunci bilaterally | Multifocal (brainstem, C6-T2 spinal cord segments, +-forebrain) |
| 53 | Fisher et al., 2024 | UK | Labrador Retriever (ME) | 1.8 | NA | NA | NA | Ambulatory paraparesis | T3-L3 spinal cord segments |
| 54 | Fisher et al., 2024 | UK | Labrador Retriever (FE) | 0.4 | NA | NA | NA | Ambulatory tetraparesis | C1-C5 spinal cord segments |
| 55 | Fisher et al., 2024 | UK | Greyhound (MN) | 6 | NA | NA | NA | Ambulatory generalised ataxia, tetraparesis, generalised muscle wastage, positional nystagmus | Multifocal CNS and PNS (brainstem and myopathy) |
| 56 | Fisher et al., 2024 | UK | Lurcher (ME) | 6 | NA | NA | NA | Ambulatory generalised ataxia, tetraparesis, right-sided head tilt, crossed-extensor reflex in the pelvic limbs, mydriasis OD with absent direct PLR OD | Multifocal (brainstem and T3-L3 spinal cord segments) |
| 57 | Fisher et al., 2024 | UK | Greyhound (MN) | 6 | NA | NA | NA | Ambulatory generalised ataxia, tetraparesis, muscle atrophy left pelvic limb, reduced pelvic limb withdrawals, absent perineal reflex, absent patellar reflexes | Multifocal CNS and PNS (myelopathy, neuropathy, myopathy) |
| 58 | Fisher et al., 2024 | UK | Greyhound (MN) | 10 | NA | NA | NA | Ambulatory generalised ataxia, hypermetric gait, wide-based stance, intention tremors, mild head tilt (to the right) | Cerebellum |
| 59 | Fisher et al., 2024 | UK | Doberman Pinscher (ME) | 7 | NA | NA | NA | Ambulatory generalised ataxia, obtundation, left-sided head tilt, reduced menace OS, positional nystagmus | Multifocal (forebrain and brainstem) |
| 60 | Fisher et al., 2024 | UK | Labrador Retriever (MN) | 3 | NA | NA | NA | Ambulatory LMN paraparesis, stiff pelvic limb gait, reduced pelvic limb withdrawals and anal tone | L4-S3 spinal cord segments |
| 61 | Fisher et al., 2024 | UK | Labrador Retriever (MN) | 6 | NA | NA | NA | Ambulatory with a short strided stiff gait in all limbs, generalised muscle atrophy (particularly affecting the masticatory muscles), moderately reduced withdrawals all limbs, reduced patellar reflex bilaterally | PNS  Neuromuscular |
| 62 | Fisher et al., 2024 | UK | Labrador Retriever (MN) | 8 | NA | NA | NA | Ambulatory generalised ataxia, hypermetric gait, intention tremor | Cerebellum |
| 63 | Fisher et al., 2024 | UK | Old English Sheepdog (FN) | 4 | NA | NA | NA | Ambulatory generalised ataxia, hypermetric gait, absent menace response OU | Cerebellum |
| 64 | Fisher et al., 2024 | UK | Cavalier King Charles Spaniel (MN) | 6 | NA | NA | NA | Seizures, absent menace OS, reduced menace OD | Forebrain |
| 65 | Fisher et al., 2024 | UK | Labrador Retriever (MN) | 9 | NA | NA | NA | Generalised ataxia and ambulatory paraparesis, absent proprioceptive responses pelvic limbs, delayed proprioceptive responses thoracic limbs (right-lateralised), reduced withdrawal reflexes pelvic limbs, reduced patellar reflexes bilaterally | Multifocal CNS and PNS (myelopathy and neuropathy/myopathy) |
| 66 | Fisher et al., 2024 | UK | French Bulldog (ME) | 0.3 | NA | NA | NA | Ambulatory generalised ataxia, equivocal obtundation, spontaneous nystagmus (rotatory), absent menace OD | Multifocal (forebrain and brainstem) |
| 67 | Fisher et al., 2024 | UK | Irish Setter (ME) | 0.8 | NA | NA | NA | Ambulatory LMN tetraparesis, generalised muscle wastage, decreased withdrawals all 4 limbs | PNS  Neuromuscular |
| 68 | Albertini et al., 2022 | UK | Greyhound (MN) | 11 | 2 weeks, progressive | Progressive non-painful ambulatory paraparesis, rapidly deteriorating to non-ambulatory tetraparesis after 48 hours | Cachexia (body condition score 3/9) and increased respiratory efforts with moderate abdominal breathing without any abnormalities on lung auscultation could be noticed. | Non-ambulatory tetraparesis, mild right-sided head tilt, marked vestibulocerebellar ataxia and a tendency to fall to the right. Postural reactions were absent in the four limbs. Absent menace response bilaterally, an absent nasal mucosa sensation response bilaterally, a positional rotatory nystagmus | Multifocal intracranial |
| 69 | Lopes et al., 2022 | UK | Greyhound (MN) | 2 | 6 weeks, progressive | Progressive generalized vestibulocerebellar ataxia starting with pelvic limb weakness that quickly progressed to ambulatory tetraparesis and vestibulocerebellar ataxia. Visual deficits and behavioral changes (anxiety) were also reported during the progression. | Unremarkable | Wide-based stance and generalized vestibulocerebellar ataxia. Postural reactions delayed on all four limbs. Absent menace response bilaterally. | Multifocal (forebrain and cerebellum) |
| 70 | Kennedy et al., 2024 | Australia | Pug (FN) | 10 | 2 weeks | Subtle behaviour changes - not eating | NA | NA | PNS - myopathy |
| 71 | Kennedy et al., 2024 | Australia | Cavalier King Charles Spaniel (FN) | 5 | 5 weeks | NA | NA | Cervical myelopathy, head/neck myoclonus | Multifocal |
| 72 | Kennedy et al., 2024 | Australia | Hungarian vizsla (ME) | 0.75 | 10 days | NA | NA | Proprioceptive deficits on the right side, subtle head tilt to the right, long stride gait, mild hind limb ataxia, muscle atrophy temporalis right side | Multifocal |
| 73 | Kennedy et al., 2024 | Australia | French Bulldog (MN) | 0.83 | 4 days | Progressive flaccid paralysis | NA | Flaccid paralysis | PNS – neuropathy |
| 74 | Kennedy et al., 2024 | Australia | Cross breed (cocker spaniel x poodle) (FN) | 10 | 2 weeks | NA | NA | Generalised marked cerebellar ataxia, hypermetric in left front and left hind limbs, mild right head tilt | Cerebellum |
| 75 | Kennedy et al., 2024 | Australia | Pug (MN) | 1 | 3 weeks | NA | NA | Paraparesis, brisk forelimb reflexes, right front limb paresis, postural deficit in the right front limb | CNS – cervical spinal cord |
| 76 | Kennedy et al., 2024 | Australia | Cavalier King Charles Spaniel (FN) | 5 | 2 weeks | NA | NA | Non-ambulatory vestibular generalised ataxia, absent menace OS, hyperaesthetic facial sensation | Multifocal |
| 77 | Kennedy et al., 2024 | Australia | Cross breed (Labrador x poodle) (MN) | 0.67 | 3 weeks | weakness --> progressive flaccid tetraparesis | Pyrexia (39.4) | Flaccid tetraparesis | PNS – neuropathy |
| 78 | Kennedy et al., 2024 | Australia | WHWT (FN) | 1.5 | 1 week | NA | NA | Mildly altered (quiet) mentation, mild generalized ataxia and hypermetric forelimbs, inconsistent menace, proprioceptive deficits pelvic limbs | Multifocal |
| 79 | Kennedy et al., 2024 | Australia | WHWT (FN) | 4 | 12 weeks | Abnormal gait | NA | Tetraparesis, poor proprioception and loss of balance | Multifocal |
| 80 | Kennedy et al., 2024 | Australia | Greyhound (FN) | 4 | 12 weeks | Pelvic limb ataxia and paresis | NA | Pelvic limb ataxia and paresis | Spinal cord segments caudal to L3 |
| 81 | Kennedy et al., 2024 | Australia | Greyhound (MN) | 4 | 3 days | NA | NA | Non-ambulatory generalised ataxia and paresis, seizures | Multifocal: CNS and PNS (myopathy) |
| 82 | Kennedy et al., 2024 | Australia | Greyhound (FN) | 9 | 1 week | Progressive tetraparesis | NA | Tetraparesis, central vestibular / brainstem signs | Multifocal CNS |

Legend:

FE – female entire, FN – female neutered, ME – male entire, MN – male neutered, NA – not available, WHWT – West Highland White Terrier, UK – United Kingdom.

**Table S2. CSF examination, serum biochemistry, serology and CSF PCR results**

| **Case Number** | **Author and Year of Publication** | **CSF Abnormalities (TNCC (cells/microL) and Protein Concentration (g/L))** | **CSF cytology** | **Serum Biochemistry Abnormalities** | **Serology Titers (Blood)** | **Follow-up Serology Titers (Time point after diagnosis)** | **CSF PCR** |
| --- | --- | --- | --- | --- | --- | --- | --- |
| 1 | Gaitero et al., 2006 | TNCC 1450  PC: 9.92 | Severe mixed cell pleocytosis (predominantly mononuclear; Intracellular and extracellular protozoal tachyzoites detected) | Hypergammaglobulinemia (2.03 g/dL) | NA | NA | NA |
| 2 | Garosi et al., 2010 | NA (TNCC range: 12–300, PC range 0.6–2.9) | Mononuclear pleocytosis | NA | ≥ 1:800 | NA | Positive |
| 3 | Garosi et al., 2010 | NA (TNCC range: 12–300, PC range 0.6–2.9) | Mononuclear pleocytosis | NA | ≥ 1:800 | NA | Negative |
| 4 | Garosi et al., 2010 | NA (TNCC range: 12–300, PC range 0.6–2.9) | Mononuclear pleocytosis | NA | ≥ 1:800 | NA | Positive |
| 5 | Garosi et al., 2010 | NA (TNCC range: 12–300, PC range 0.6–2.9) | Mononuclear pleocytosis | NA | ≥ 1:800 | NA | Positive |
| 6 | Garosi et al., 2010 | Unremarkable | Unremarkable | NA | ≥ 1:800 | NA | Positive |
| 7 | Garosi et al., 2010 | NA (TNCC range: 12–300, PC range 0.6–2.9) | Mononuclear pleocytosis | NA | ≥ 1:800 | NA | NA |
| 8 | Garosi et al., 2010 | NA (TNCC range: 12–300, PC range 0.6–2.9) | Mononuclear pleocytosis | NA | NA | NA | NA |
| 9 | Parzefall et al., 2014 | Unremarkable | Macrophagic inflammation with unremarkable TNCC and unremarkable PC | CK 3033 U/L, ALT 317 U/L, Bilirubin 4.0 µmol/L, AST not provided | > 1:1600 | NA | Positive |
| 10 | Parzefall et al., 2014 | TNCC 48  PC: 0.34 | Mixed cell pleocytosis | CK 1507 U/L, ALT 159 U/L, AST not provided | > 1:1600 | NA | Positive |
| 11 | Parzefall et al., 2014 | Cisternal:  TNCC 137  PC 0.94  Lumbar:  TNCC 168  PC 1.73 | Neutrophilic pleocytosis | CK 897 U/L, ALT 95 U/L, AST not provided | > 1:1600 | NA | Positive |
| 12 | Parzefall et al., 2014 | TNCC 282  PC 0.42 | Mixed cell pleocytosis | CK 1729 U/L, ALT 135 U/L, AST not provided | > 1:800 | Serology remained negative 3, 6, and 10 months after diagnosis | Positive |
| 13 | Coelho et al., 2018 | NA | Mixed cell pleocytosis | NA | 1:800 | NA | Positive |
| 14 | Coelho et al., 2018 | NA | Mixed cell pleocytosis | NA | NA | NA | Positive |
| 15 | Coelho et al., 2018 | NA | Mixed cell pleocytosis | NA | 1:800 | NA | Negative |
| 16 | Coelho et al., 2018 | NA | Pleocytosis (cytology NA) | NA | 1:1600 | NA | NA |
| 17 | Coelho et al., 2018 | NA | Mixed cell pleocytosis | NA | 1:3200 | NA | NA |
| 18 | Coelho et al., 2018 | NA | Mononuclear pleocytosis | NA | 1:800 | NA | NA |
| 19 | Coelho et al., 2018 | NA | Albuminocytological dissociation | NA | 1:800 | NA | NA |
| 20 | Coelho et al., 2018 | NA | Mononuclear pleocytosis | NA | 1:800 | NA | NA |
| 21 | Coelho et al., 2018 | NA | Mononuclear pleocytosis | NA | 1:1600 | NA | NA |
| 22 | Didiano et al., 2020 | TNCC 30  PC 0.61 | Neutrophilic pleocytosis | ALT (277 U/L), CK (3777 U/L), AST (202 IU/L), cholesterol (8.7 mmol/L), triglyceride (1.4 mmol/L) | 1:3200 | 1:800 (after 8 weeks)  1:800 (after 6 months) | Positive |
| 23 | Didiano et al., 2020 | TNCC 13  PC 0.85 | Eosinophilic pleocytosis | CK (1494 IU/L), ALT and AST unremarkable | > 1:800 | 1:400 (after 2 months)  1:400 (after 12 weeks) | Positive |
| 24 | Didiano et al., 2020 | TNCC 46  PC 0.85 | Mononuclear pleocytosis | Unremarkable CK, ALT, AST | 1:800 | Unchanged 1:800 (after 6 months) | Positive |
| 25 | Didiano et al., 2020 | TNCC unremarkable  PC 0.39 | Albuminocytological dissociation | Unremarkable CK, ALT, AST | > 1:800 | Unchanged ≥ 1:800 (after 6 months) | Positive |
| 26 | Didiano et al., 2020 | TNCC 7  PC 0.84 | Eosinophilic pleocytosis | CK (1169 IU/L), unremarkable ALT, AST | > 1:800 | Unchanged ≥ 1:800 (after 4 months) | Positive |
| 27 | Didiano et al., 2020 | TNCC 17  PC 0.47 | Neutrophilic pleocytosis | ALT (678 IU/L), AST (471 IU/L), CK (8383 IU/L) | > 1:800 | Increased 1:1600 (after 8 weeks)  1:100 (after 6 and 7 months) | Positive |
| 28 | Clark et al., 2022 | TNCC 72  PC 1.39 | Eosinophilic pleocytosis | CK 1747 U/L, AST 102 U/L, ALT (128 U/L), ALP (123 U/L), phosphate (2.4 mmol/L), cholesterol (8.9 mmol/L) | 1:400 | Rising to 1:3200 (after 10 days) | Negative |
| 29 | Clark et al., 2022 | TNCC 15  PC 0.99 | Neutrophilic pleocytosis | CK 2652 U/L (rising to 11,584 U/L 17 days later), AST 251 U/L, ALT 434 U/L, GLDH 6.2 U/L; proteinuria 16,241.0 mg/L with a urine UPC ratio of 7.31 | >1:800 | NA | Positive |
| 30 | Alf et al., 2024 | NA | NA | NA | **NA** | NA | NA |
| 31 | Alf et al., 2024 | NA | NA | ALT 854 U/L, CK 14,062 U/L, CRP 16.7 mg/L, AST unremarkable | 1:320 | Negative on day 2, remained the same 1:320 after 2 weeks and 11 weeks | NA |
| 32 | Alf et al., 2024 | NA | NA | ALT 293 U/L, CK 6,939 U/L, AST 311 U/L | 1:640 | **NA** | NA |
| 33 | Fisher et al., 2024 | NA | NA | CK 43 microkat/L (unremarkable), AST 29 U/L (unremarkable) | 1:1600 | NA | NA |
| 34 | Fisher et al., 2024 | TNCC 0  PC increased | Albuminocytological dissociation | CK 107 U/L (unremarkable), AST 25 U/L (unremarkable) | >1600 | NA | Negative |
| 35 | Fisher et al., 2024 | TNCC 0 | Unremarkable | CK 136 U/L (unremarkable), AST 39 U/L (unremarkable) | >12560 | NA | Negative |
| 36 | Fisher et al., 2024 | TNCC 0 | Unremarkable | CK 187 U/L (unremarkable), AST 35 U/L (unremarkable) | 6400 | NA | Negative |
| 37 | Fisher et al., 2024 | TNCC 7 | Mixed cell pleocytosis | CK 281 U/L, AST 50 U/L (unremarkable) | >1600 | NA | Negative |
| 38 | Fisher et al., 2024 | TNCC 9 | Mononuclear pleocytosis (lymphocytic predominance) | CK 513 U/L, AST 88 U/L | 1600 | NA | Negative |
| 39 | Fisher et al., 2024 | TNCC 70 | Mononuclear pleocytosis | CK 521 U/L, AST 66 U/L | >1600 | NA | Positive |
| 40 | Fisher et al., 2024 | TNCC 70 | Neutrophilic pleocytosis | CK 560 U/L, AST 59 U/L | >1600 | NA | Positive |
| 41 | Fisher et al., 2024 | TNCC 13 | Neutrophilic pleocytosis | CK 682 U/L, AST 59 U/L | 800 | NA | Positive |
| 42 | Fisher et al., 2024 | TNCC 7 | Eosinophilic pleocytosis | CK 755 U/L, AST 128 U/L | 1600 | Serology repeated at 1.8, 10.1, 11.8 months after diagnosis – all in normal range at 11.9 months after diagnosis (unknown at other time points) | Positive |
| 43 | Fisher et al., 2024 | TNCC 57 | Eosinophilic pleocytosis | CK 783 U/L, AST 120 U/L | >1600 | NA | Positive |
| 44 | Fisher et al., 2024 | TNCC 11 | Neutrophilic pleocytosis | CK 1261 U/L, AST 145 U/L | >800 | NA | NA |
| 45 | Fisher et al., 2024 | TNCC 4 | Albuminocytological dissociation | CK 1281 U/L, AST 141 U/L | >1600 | >1600 (at first relapse (after 8.3 months), 51200 (at second relapse (unknown date)) | Positive |
| 46 | Fisher et al., 2024 | TNCC 160 | Mononuclear pleocytosis (lymphocyte predominance) | CK 1327 U/L, AST 80 U/L | >1600 | >1600 (at relapse after 16 months) | Positive |
| 47 | Fisher et al., 2024 | TNCC 40 | Mononuclear pleocytosis (lymphocyte predominance) | CK 1332 U/L, AST 105 U/L | >1600 | NA | Negative |
| 48 | Fisher et al., 2024 | TNCC 25 | Mixed cell pleocytosis (mononuclear predominance) | CK 1423 U/L, AST 154 U/L | 800 | NA | Positive |
| 49 | Fisher et al., 2024 | TNCC 77 | Mixed cell pleocytosis (lymphocyte predominance) | CK 1745 U/L, AST 161 U/L | >1600 | NA | Negative |
| 50 | Fisher et al., 2024 | TNCC 20 | Mononuclear pleocytosis | CK 2024 U/L, AST 128 U/L | >800 | NA | NA |
| 51 | Fisher et al., 2024 | TNCC 0 | Albuminocytological dissociation | CK 2062 U/L, AST 237 U/L | >1600 | NA | Positive |
| 52 | Fisher et al., 2024 | TNCC 102 | Eosinophilic pleocytosis | CK 2173 U/L, AST 119 U/L | >1600 | 1:800 (at relapse after 4.5 months) | Positive |
| 53 | Fisher et al., 2024 | TNCC 2 | Albuminocytological dissociation | CK 2857 U/L, AST 211 U/L | >1600 | NA | Negative |
| 54 | Fisher et al., 2024 | TNCC 30 | Mononuclear pleocytosis (lymphocytic predominance) | CK 3482 U/L, AST 167 U/L | >1600 | NA | Negative |
| 55 | Fisher et al., 2024 | TNCC 15 | Mixed cell pleocytosis | CK 3500 U/L, AST 333 U/L | >1600 | NA | Negative |
| 56 | Fisher et al., 2024 | TNCC 0 | Albuminocytological dissociation | CK 3633 U/L, AST 160 U/L | >3200 | NA | Negative |
| 57 | Fisher et al., 2024 | TNCC 3 | Albuminocytological dissociation | CK 3823 U/L, AST 325 U/L | >1600 | NA | Negative |
| 58 | Fisher et al., 2024 | TNCC 25 | Mild mixed cell pleocytosis | CK 4447 U/L, AST 273 U/L | 400 | NA | Positive |
| 59 | Fisher et al., 2024 | TNCC 0 | Albuminocytological dissociation | CK 5020 U/L, AST not performed | >1600 | 1:800 (after 2.6 months) 1:200 (after 6.2 months (neurologically stable)) | Negative |
| 60 | Fisher et al., 2024 | TNCC 53 | Mononuclear pleocytosis (lymphocyte predominance) | CK 6365 U/L, AST 337 U/L | >1600 | NA | Negative |
| 61 | Fisher et al., 2024 | NA | NA | CK 9631 U/L, AST >1083 U/L | 800 | NA | NA |
| 62 | Fisher et al., 2024 | TNCC 7 | Eosinophilic pleocytosis | CK Elevated (over measurable range), AST 190 U/L | >800 | NA | NA |
| 63 | Fisher et al., 2024 | TNCC 0 | Albuminocytological dissociation | CK not performed, AST not performed | 1600 | NA | Negative |
| 64 | Fisher et al., 2024 | TNCC 21 | Mononuclear pleocytosis (lymphocyte predominance) | CK not performed, AST not performed | 800 | NA | Negative |
| 65 | Fisher et al., 2024 | TNCC 20 | Eosinophilic pleocytosis | CK not performed, AST not performed | >800 | NA | Positive |
| 66 | Fisher et al., 2024 | TNCC 7 | Mononuclear pleocytosis (lymphocyte predominance) | CK not performed, AST not performed | >1600 | NA | Negative |
| 67 | Fisher et al., 2024 | TNCC 20 | Mixed cell pleocytosis | CK not performed, AST not performed | >1600 | NA | NA |
| 68 | Albertini et al., 2022 | TNCC 13  PC 1.02 | Eosinophilic pleocytosis | ALT: 265 U/L | 1:400 (IFT)  *Toxoplasma gondii* serology positive for IgG at 1:100 and negative for IgM | NA | Positive |
| 69 | Lopes et al., 2022 | TNCC 11  PC 0.37 | Neutrophilic pleocytosis | ALT: 280 U/L  AST 145 U/L  CK 1261 U/L  AST not performed | ≥ 1:800 | NA | NA |
| 70 | Kennedy et al., 2024 | NA | NA | ALT 247 U/L  CK 729 U/L  AST not performed | 1:1600  Toxoplasma titer 1:4096 | NA | NA |
| 71 | Kennedy et al., 2024 | Lumbar: TNCC 0  PC 0.6 | Albuminocytological dissociation | ALT 280 U/L  CK 2420 U/L  AST not performed | 1:6400 | 1:50 (after 15 months) | Negative |
| 72 | Kennedy et al., 2024 | TNCC 124  PC 2.17 | Eosinophilic pleocytosis | ALT 440 U/L  CK 3369 U/L  AST not performed | 1:3200 | NA | Negative |
| 73 | Kennedy et al., 2024 | NA | NA | ALT 16815 U/L  CK 30126 U/L  AST not performed | 1:800 | NA | NA |
| 74 | Kennedy et al., 2024 | NA | NA | ALT 196 U/L  CK not performed  Globulins 48 g/L  AST not performed | 1:12800 | NA | NA |
| 75 | Kennedy et al., 2024 | NA | NA | ALT 152 U/L  CK not performed  AST not performed | 1:25000  Toxoplasma titer 1:128 | NA | NA |
| 76 | Kennedy et al., 2024 | NA | NA | ALT unremarkable  CK not performed  AST not performed | 1:3200 | NA | NA |
| 77 | Kennedy et al., 2024 | NA | NA | ALT 483 U/L  CK not performed  AST not performed | 1:3200 | NA | NA |
| 78 | Kennedy et al., 2024 | TNCC 41 | Mixed cell pleocytosis (predominantly monocytic) | Globulins 52 g/L  ALT 256 U/L  CK not performed  AST not performed | 1:6400 | NA | NA |
| 79 | Kennedy et al., 2024 | NA | NA | CK 422 U/L  AST not performed  ALT unremarkable | 1:6400 | NA | NA |
| 80 | Kennedy et al., 2024 | Lumbar: blood contamination  NA | NA | ALT 187 U/L  CK not performed  AST not performed | 1:12800  Toxoplasma titer positive 1:128 | NA | NA |
| 81 | Kennedy et al., 2024 | TNCC 6.6  PC 0.36 | Mixed cell pleocytosis (predominantly macrophages) | ALT 1264 U/L  CK 360154 U/L  AST not performed | 1:12800 | NA | Negative |
| 82 | Kennedy et al., 2024 | TNCC 80  PC 2.87 | Mixed cell pleocytosis (predominantly monocytic) | ALT 783 U/L  T BIL 18  CK 1112 U/L  AST not performed | 1:6400 | NA | Positive |

Legend:

TNCC – total nucleated cell count

PC – protein concentration

NA – not available

**Table S3. MRI findings**

| **Nr** | **Author** | **Neurological localisation** | **CNS region undergoing imaging** | **MRI findings** | **MRI lesion distribution** | **Contrast enhancement** | **White/grey matter affected** | **Muscles affected** | **T2w/FLAIR hyperintense lesion surrounding cerebellum** |
| --- | --- | --- | --- | --- | --- | --- | --- | --- | --- |
| 1 | Gaitero et al., 2006 | Diffuse/multifocal (forebrain and brainstem) | Brain | Leptomeningeal thickening with diffuse meningeal contrast enhancement. Focal cortical-subcortical parietotemporal lesion homogeneously hyperintense in T2 images causing mass effect, not contrast enhancing. | Focal forebrain | Yes – meningeal | Both | No | No |
| 2 | Garosi et al., 2010 | Multifocal (cerebellum and brainstem) | Brain | Cerebellar atrophy, contrast enhancement of the affected meninges. The region surrounding the atrophied cerebellum remained hyperintense on FLAIR sequences, contrast enhancing. | Diffuse cerebellar | Yes - meningeal | NA | No | Yes |
| 3 | Garosi et al., 2010 | Cerebellum | Brain | Cerebellar atrophy, T2 and FLAIR hyperintense SI in cerebellar white matter, bilaterally symmetrical T2-weighted and FLAIR hyperintensities in the corona radiata of the occipital lobes. The region surrounding the atrophied cerebellum remained hyperintense on FLAIR sequences. | Multifocal forebrain, cerebellum | No | Both | No | Yes |
| 4 | Garosi et al., 2010 | Multifocal (cerebellum and brainstem) | Brain | Cerebellar atrophy, T2 and FLAIR hyperintense SI in cerebellar white matter. The region surrounding the atrophied cerebellum remained hyperintense on FLAIR sequences. | Multifocal/diffuse cerebellar | No | Both | No | Yes |
| 5 | Garosi et al., 2010 | Multifocal (cerebellum, brainstem, and L4-S3 spinal cord) | Brain and spine | Cerebellar atrophy, T2 and FLAIR hyperintense SI in cerebellar white matter. The region surrounding the atrophied cerebellum remained hyperintense on FLAIR sequences. Heterogenous T2-weighted hyperintensities and contrast enhancement of the temporalis and masseter muscles, which appeared moderately atrophied. Lumbar spine was unremarkable. | Diffuse cerebellar  Masticatory muscles | Yes - intramuscular | Both | Yes | Yes |
| 6 | Garosi et al., 2010 | Multifocal (cerebellum and brainstem) | Brain | Cerebellar atrophy. The region surrounding the atrophied cerebellum remained hyperintense on FLAIR sequences. Heterogenous T2-weighted hyperintensities and contrast enhancement of the temporalis and masseter muscles, which appeared moderately atrophied. | Diffuse cerebellar  Masticatory muscles | Yes - intramuscular | NA | Yes | Yes |
| 7 | Garosi et al., 2010 | Multifocal (cerebellum and brainstem) | Brain | Cerebellar atrophy, Contrast enhancement of the affected meninges. The region surrounding the atrophied cerebellum remained hyperintense on FLAIR sequences, contrast enhancing. Heterogenous T2-weighted hyperintensities and contrast enhancement of the temporalis and masseter muscles, which appeared moderately atrophied. | Diffuse cerebellar  Masticatory muscles | Yes – meningeal  Yes - intramuscular | NA | Yes | Yes |
| 8 | Garosi et al., 2010 | Multifocal (cerebellum and brainstem) | Brain | Cerebellar atrophy. | Diffuse cerebellar | No | NA | No | No |
| 9 | Perzefall et al., 2014 | Multifocal brain (with left prosencephalic component) | Brain | Multifocal intra-axial ill-defined lesions in thalamus, internal capsule, centrum semiovale, and in the gray matter of the parietal and temporal lobes. The lesions were hyperintense on T2-weighted and FLAIR images and iso-hypointense on T1-weighted images with mild peripheral contrast enhancement. | Multifocal forebrain | Yes – intraparenchymal brain | Both | No | No |
| 10 | Perzefall et al., 2014 | Multifocal (forebrain and cerebellum) | Brain | Cerebellar atrophy. An extensive intra-axial, poorly demarcated mesencephalic and metencephalic midline lesion. The lesion was hyperintense on T2-weighted and FLAIR images and iso-hypointense on T1-weighted with mild contrast enhancement. | Multifocal brainstem, cerebellum | Yes – intraparenchymal brain | Both | No | No |
| 11 | Perzefall et al., 2014 | Multifocal (C1-T2, L4-S3 spinal cord segments and cerebellum) | Brain and spine | Cerebellar atrophy. Multifocal ill-defined intramedullary spinal cord lesions at the level of C5 and C6 affecting both ventral horns as well as lateral and ventral funiculi. These spinal lesions were hyperintense on T2-weighted and isointense on T1-weighted images with no contrast enhancement. | Multifocal cerebellum, spinal cord | No | Both | No | No |
| 12 | Perzefall et al., 2014 | Multifocal CNS disease | Brain and spine | Cerebellar atrophy. Multifocal intra-axial lesions affecting the thalamus, lentiform nucleus, centrum semiovale, mesencephalon, metencephalon, subcortical grey matter of the frontal, parietal, and temporal lobes and an intramedullary spinal cord lesion mainly affecting the right dorsal horn at the level of C2-C3. The lesions were hyperintense on T2-weighted and FLAIR images and iso-hypointense on T1 with minimal contrast enhancement. | Multifocal cerebellum, forebrain, brainstem, spinal cord | Yes – intraparenchymal brain  Yes – intraparenchymal spinal cord | Both | No | No |
| 13 | Coelho et al., 2018 | Multifocal | Brain | Cerebellar atrophy | Diffuse cerebellar | NA | NA | NA | NA |
| 14 | Coelho et al., 2018 | Cerebellum | Brain | Cerebellar atrophy | Diffuse cerebellar | NA | NA | NA | NA |
| 15 | Coelho et al., 2018 | Multifocal | Brain | Multifocal | Multifocal unknown areas | NA | NA | NA | NA |
| 16 | Coelho et al., 2018 | Multifocal | Brain | Cerebellar atrophy | Diffuse cerebellar | NA | NA | NA | NA |
| 17 | Coelho et al., 2018 | Multifocal | Brain | Multifocal | Multifocal unknown areas | NA | NA | NA | NA |
| 18 | Coelho et al., 2018 | Multifocal | Brain | Multifocal | Multifocal unknown areas | NA | NA | NA | NA |
| 19 | Coelho et al., 2018 | Cerebellum | Brain | Cerebellar atrophy | Diffuse cerebellar | NA | NA | NA | NA |
| 20 | Coelho et al., 2018 | Forebrain | Brain | Multifocal | Multifocal unknown areas | NA | NA | NA | NA |
| 21 | Coelho et al., 2018 | Multifocal | Brain | Cerebellar atrophy | Diffuse cerebellar | NA | NA | NA | NA |
| 22 | Didiano et al., 2020 | Cerebellum | Brain | Cerebellar atrophy, with increased signal intensity (SI) on T2-weighted and T2 FLAIR images, primarily affecting the cerebellar cortex. Increased CSF accumulation between the cerebellar folia. No contrast enhancement observed. | Diffuse cerebellar | No | Grey | No | Yes |
| 23 | Didiano et al., 2020 | T3–L3 spinal cord segment | Spine | MRI of the thoracolumbar spine was unremarkable. | None | No | None | No | NA |
| 24 | Didiano et al., 2020 | C1–C5 spinal cord segment | Brain and spine | MRI revealed multifocal, intra-axial lesions with increased T2 and STIR signal intensities at C3, C5, and C6–C7. There was focal but marked contrast enhancement at C3. T2W and FLAIR increased SI around the mesencephalon and cerebellum, contrast enhancing. | Multifocal spinal cord, cerebellum, brainstem | Yes – meningeal  Yes – intraparenchymal spinal cord and brain | NA | No | Yes |
| 25 | Didiano et al., 2020 | Multifocal (cerebellum and forebrain) | Brain | Cerebellar atrophy. Heterogeneous T2W and FLAIR hyperintensities were noted in the thalamus and rostral brainstem. | Multifocal cerebellar, forebrain, brainstem | No | Both | No | No |
| 26 | Didiano et al., 2020 | Cerebellum | Brain | Cerebellar atrophy. | Focal cerebellar | No | Grey | No | No |
| 27 | Didiano et al., 2020 | Multifocal intracranial (cerebellum and brainstem) | Brain | Multifocal ill-defined T1W isointense, T2W and FLAIR hyperintense areas involving the right caudate nucleus, right frontal lobe cortex, left hippocampus, and several lesions in the medulla oblongata. Faint contrast enhancement within hippocampus and medulla oblongata was observed. | Multifocal forebrain, brainstem | Yes – intraparenchymal brain | Both | No | No |
| 28 | Clark et al., 2022 | L4–Cd spinal cord segments | Spine | Multifocal bilateral asymetric, ill-defined, patchy intramuscular lesions affecting the interspinal, middle gluteal, psoas and lumbar iliocostal muscles. Lesions are hyperintense on T2W and fat suppression images, isointense on T1W pre- contrast images, and variably moderately contrast enhancing. Bilateral, relatively symmetrical thickening of the L6 and L7 nerve roots extending into the intervertebral foramina. The left L5 nerve root and spinal nerve also appeared slightly enlarged. The enlarged nerve roots and spinal nerves were slightly hyperintense to muscles on T2W images, isointense on T1W images and moderately uniformly enhancing. There was meningeal enhancement in the region of the L4–L7 vertebrae. The medial iliac lymph nodes were mildly asymmetrically enlarged. | Multifocal nerve roots  Epaxial musculature | Yes - intramuscular  Yes – meningeal  Yes - nerve roots | None | Yes | NA |
| 29 | Clark et al., 2022 | L4–S1 spinal cord segments, nerve roots or spinal nerves | Spine | A single, poorly defined intramedullary hyperintense on T2W, isointense to normal SI on T1W sequences and without contrast enhancement lesion overlying the L4 vertebra. It’s predominantly affecting the grey matter on the right side. There was mild focal swelling of the spinal cord. Mild, patchy meningeal contrast enhancement in the region of the L4 vertebra to the conus medullaris. On fat suppression images, there were multiple, poorly defined, patchy hyperintense lesions throughout the paraspinal muscles and sartorius and middle gluteal muscles, which were bilaterally asymmetrically distributed, and displayed mild patchy contrast enhancement. | Multifocal spinal  Epaxial musculature | Yes – meningeal  Yes - intramuscular | Both | Yes | NA |
| 30 | Alf et al., 2024 | Multifocal: CNS and PNS | Brain | Multifocal intra-axial lesions of the brain compatible with encephalitis. | Multifocal brain unknown areas | NA | NA | No | NA |
| 31 | Alf et al., 2024 | Multifocal: CNS and PNS | Brain | Cerebellar atrophy, hyperintense rim of the subdural space around the cerebellum in the T2W and FLAIR sequences, it’s hypointense/isointense in T1W images with marked contrast enhancement. Moreover, the right masseter muscle appeared markedly atrophic, and the entire masticatory muscles showed significant contrast enhancement. | Diffuse cerebellar  Masticatory muscles | Yes – meningeal  Yes – intraparenchymal brain  Yes - intramuscular | Grey | Yes | Yes |
| 32 | Alf et al., 2024 | Multifocal: CNS and PNS | Brain | Multiple intra-axial lesions in the brain. | Multifocal brain unknown areas | NA | NA | NA | NA |
| 33 | Fisher et al., 2024 | T3-S1 spinal cord segments | Spine | Unremarkable. | None | No | None | No | NA |
| 34 | Fisher et al., 2024 | T3-L3 spinal cord segments | Spine | Right-sided focal poorly defined hyperintensity in right lateral funiculus of L2-L3 SCS. | Focal spinal | No | White | No | NA |
| 35 | Fisher et al., 2024 | Forebrain | Brain | Unremarkable. | None | No | None | No | No |
| 36 | Fisher et al., 2024 | Multifocal (forebrain and cerebellum) | Brain | Poorly-defined focal area of T2W and FLAIR hyperintensity within the white matter of the right temporal lobe, symmetrical mild dilation of lateral ventricles. | Focal forebrain | No | White | No | No |
| 37 | Fisher et al., 2024 | T3-L3 spinal cord segments | Brain and spine | Hypoplastic/atrophic thoracolumbar spinal cord and cerebellar atrophy. | Multifocal cerebellar and spinal cord | No | None | No | No |
| 38 | Fisher et al., 2024 | Multifocal (forebrain and brainstem) | Brain | T2W hyperintense lesion in midbrain (extending to thalamus and brainstem), reactive right medial retropharyngeal lymph node. | Multifocal forebrain and brainstem | No | NA | No | No |
| 39 | Fisher et al., 2024 | Multifocal (T3-L3 spinal cord segments and L6-S1 spinal cord segments /sciatic neuropathy) | Spine | Unremarkable. | None | No | None | No | NA |
| 40 | Fisher et al., 2024 | C1-C5 spinal cord segments | Brain and spine | Streaky contrast enhancement of muscles of head bilaterally and marbled on FLAIR  Multiple degenerative spinal discs without spinal cord compression (C6-7, T4-8, T12-13), spinal cord unremarkable. | Masticatory muscles | Yes – intramuscular | None | Yes | No |
| 41 | Fisher et al., 2024 | T3-L3 spinal cord segments | Spine | Patchy T2 hyperintense lesions in L3-L6 and C6-T2 cord, mild contrast enhancement of lesions. | Multifocal spinal cord | Yes – intraparenchymal spinal cord | NA | No | No |
| 42 | Fisher et al., 2024 | Multifocal (forebrain and brainstem) | Brain | Multiple intra-axial T2W and FLAIR hyperintense lesions in the left cerebral cortex grey matter (adjacent to corona radiata and occipital lobe), dorso-rostral cerebellum, right lateral ventricle grey matter, and dorsal brainstem. All lesions show mild contrast enhancement. | Multifocal forebrain, cerebellum, brainstem | Yes – intraparenchymal brain | Grey | No | NA |
| 43 | Fisher et al., 2024 | Multifocal (forebrain and cerebellum) | Brain | Atrophy of cerebellum (particularly right side), contrast enhancement of meninges of dorsal cerebellum. | Diffuse cerebellar | Yes - meningeal | NA | No | NA |
| 44 | Fisher et al., 2024 | Cerebellum | Brain | Marked cerebral and cerebellar atrophy is present with thinning of the cortices, ventriculomegaly.  A focal area of increased intensity without contrast enhancement in the dorsal aspect of the right frontal sinus. | Diffuse/multifocal forebrain, cerebellum | No | Grey | No | No |
| 45 | Fisher et al., 2024 | Cerebellum | Brain | Cerebellar atrophy, T2W hyperintense halo around cerebellum with extension to sulci. | Diffuse cerebellar | No | NA | No | Yes - suspected |
| 46 | Fisher et al., 2024 | Multifocal (forebrain, brainstem, myopathy) | Brain | T2W and FLAIR focal hyperintensities in midbrain (ventrolateral aspect of mesencephalic aqueduct at level of rostral and caudal colliculi, right ventral aspect of pons, and thalamus) isointense on T1W, no contrast uptake, cerebellar atrophy, moderate ventriculomegaly, temporal muscles reduced in size bilaterally with patchy T2W and FLAIR hyperintensities with contrast enhancement. | Multifocal brainstem, forebrain, cerebellum  Masticatory muscles | Yes – intramuscular | NA | Yes | No |
| 47 | Fisher et al., 2024 | T3-L3 spinal cord segments | Brain and spine | Multifocal T2W hyperintense and T1 iso/hyperintense lesions in cranial cervical spinal cord (most marked dorsal C1-C2, grey and white matter affected) and brain (medulla oblongata, mesencephalon, right and left cerebellar peduncles), no contrast enhancement. Atrophy of cerebellum. | Multifocal spinal, brainstem, cerebellum | No | Both | No | No |
| 48 | Fisher et al., 2024 | C1-C5 spinal cord segments | Brain and spine | Suspected cerebellar atrophy. Focal, poorly-defined T2W and FLAIR hyperintense lesion on ventral aspect of pons (isointense on T1W, no contrast enhancement)  Dehydration and mild protrusion of L5-6 and L7-S1 disc, slight protrusion of L6-7 but still hydrated. Partial dehydration of C3-4 and T1-2. | Multifocal brainstem, cerebellum | No | NA | No | No |
| 49 | Fisher et al., 2024 | Multifocal (forebrain, cerebellum and neuromuscular) | Brain | Multiple ill-defined asymmetrical T2W and FLAIR hyperintense foci in grey matter of cerebral cortex (left frontal, right frontal, right temporal, left/right piriform lobes, left/right caudate nucleus) with mild contrast enhancement. Cerebellar grey matter heterogenously T2W and FLAIR hyperintensities with loss of white/grey matter distinction. | Multifocal forebrain, cerebellum | Yes – intraparenchymal brain | Grey | NA | Yes - suspected |
| 50 | Fisher et al., 2024 | Multifocal (cerebellum and T3-L3 spinal cord segments) | Brain | Cerebellar atrophy with reduced grey-white matter differentiation, cerebellar meningeal thickening and moderate contrast enhancement, possible minimal right temporal muscle atrophy. Cerebellum is surrounded by thick band of T2W and FLAIR hyperintense signal which is diffusely T1w hypointense, separating the folia of the cerebellum. | Diffuse cerebellar  Masticatory muscles | Yes - meningeal | Grey | No | Yes |
| 51 | Fisher et al., 2024 | Multifocal (forebrain and brainstem) | Brain | Multifocal T2W and FLAIR hyperintensities in brainstem at level of left facial nucleus and right caudal/medial vestibular nuclei (hypointense on T1W, mild contrast enhancement), cerebellar atrophy, T2W and FLAIR hyperintense striations in muscles of head (patchy contrast uptake), T2W, FLAIR and T1W hyperintense material in ventromedial right tympanic bulla and ventromedial/dorsal left bulla with no contrast uptake. | Multifocal brainstem, cerebellum  Masticatory muscles | Yes – intraparenchymal brain  Yes - intramuscular | Grey | Yes | No |
| 52 | Fisher et al., 2024 | Multifocal (brainstem, C6-T2 spinal cord segments, +-forebrain) | Brain | Facial and masticatory muscles T2W and FLAIR moderate to marked streaked hyperintensities (isointense on T1W, moderate to marked contrast enhancement), cerebellar atrophy. | Diffuse cerebellar  Masticatory muscles | Yes – intramuscular | Grey | Yes | No |
| 53 | Fisher et al., 2024 | T3-L3 spinal cord segments | Brain and spine | Spinal cord atrophy/hypoplasia (mid thoracic to mid lumbar), cerebellar atrophy. | Multifocal cerebellar, spinal | No | Grey | No | No |
| 54 | Fisher et al., 2024 | C1-C5 spinal cord segments | Spine | Subjective spinal cord atrophy/hypoplasia. | Diffuse spinal | No | NA | No | NA |
| 55 | Fisher et al., 2024 | Multifocal CNS and PNS (brainstem and myopathy) | Brain | Asymmetrical diffuse widening of sulci of cerebral hemispheres and cerebellar folia, marked brain atrophy with mild cerebellar atrophy, FLAIR hyperintense focal ill-defined lesions lateral to right lateral ventricle, dorsal to right piriform lobe, medial aspect of med-ectomarginal gyrus, and left lateral ventricle (no contrast enhancement). | Diffuse/multifocal forebrain, cerebellum | No | NA | No | No |
| 56 | Fisher et al., 2024 | Multifocal (brainstem and T3-L3 spinal cord segments) | Brain | Marked loss of grey and white matter of cerebellar hemispheres bilaterally, cerebellar atrophy, T2W hyperintense/T1W hypointense lesions in the cerebellum that are non-enhancing, mild ventriculomegaly of fourth ventricle. | Diffuse cerebellar | No | Both | No | No |
| 57 | Fisher et al., 2024 | Multifocal CNS and PNS (myelopathy, neuropathy, myopathy) | Spine | L3-4 and L5 left lateralized intramedullary T2W hyperintensities without contrast enhancement. | Multifocal spinal | No | NA | No | NA |
| 58 | Fisher et al., 2024 | Cerebellum | Brain | Two intra-axial well defined and partially cavitated lesions affecting the left caudate nucleus and right thalamus compatible with chronic lacunar infarcts (these areas represent the vascular territory of the striate and perforating arteries respectively), cerebellar atrophy, mild submandibular lymphadenomegaly. | Multifocal forebrain, cerebellum | No | Grey | No | No |
| 59 | Fisher et al., 2024 | Multifocal (forebrain and brainstem) | Brain | Mild cortical brain atrophy. | Diffuse brain | No | Grey | No | No |
| 60 | Fisher et al., 2024 | L4-S3 spinal cord segments | Spine | Multifocal T2W hyperintense contrast-enhancing lesions in lumbar/pelvic musculature, cauda equina nerves/meninges were contrast-enhancing, L2-L6 mild disc dehydration. | Multifocal nerves  Epaxial musculature | Yes – intramuscular  Yes – nerves  Yes - meningeal | None | Yes | NA |
| 61 | Fisher et al., 2024 | PNS  Neuromuscular | Brain | Masticatory muscles (less so – epaxial muscles) are markedly heterogeneous characterised by multiple T2W, FLAIR and STIR hyperintense linear structures that are hypointense in T2W and markedly contrast enhancing. Multifocal intra-axial ill-defined lesions are seen affecting left >right thalamus, left midbrain, left metencephalon and left myelencephalon, as well as diffusely affecting the cerebellar hemispheres mainly the left side. The lesions are hyperintense on T2W and FLAIR and iso-hypointense on T1W showing mild contrast enhancement. Mild cerebellar atrophy. | Masticatory muscles  Multifocal forebrain, brainstem, cerebellum | Yes – intramuscular  Yes – intraparenchymal brain | NA | Yes | Yes - suspected |
| 62 | Fisher et al., 2024 | Cerebellum | Brain | Cerebellar atrophy, with decreased distinction between grey and white matter. It is surrounded by thick band of T2w and FLAIR hyperintense signal which is diffusely T1W hypointense, separating the folia of the cerebellum.  Cerebral atrophy. No contrast enhancement. A well-defined rounded shape T1w hyperintense structure is present within the posterior lobe of the pituitary gland. The structure is not visible in the T2w sequences as well as DWI, and homogeneously contrast enhancing.  Mild amount of T2w hyperintense signal, T1w hypointense, partially suppressed on FLAIR, along the roof of the nasopharynx.  Incidental T2w/T1w/FLAIR hyperintense, pedunculated structure, located at the left vertical external ear canal. | Diffuse cerebellar | No | Grey | No | Yes |
| 63 | Fisher et al., 2024 | Cerebellum | Brain | Cerebellar atrophy - marked loss of grey and white matter of cerebellar hemispheres, T2W hyperintense/T1W hypointense/not fully supressed in FLAIR material surrounding cerebellum, ventriculomegaly of fourth ventricle. | Diffuse cerebellar | No | Both | No | Yes |
| 64 | Fisher et al., 2024 | Forebrain | Brain | Poorly-defined intra-axial T2W hyperintensity/T1W hypointensity of white matter at right lateral ventricle, focal T2W hyperintensity dorsal to caudal aspect of left lateral ventricle, contrast enhancing, mild dilation of central canal, crowding of posterior fossa but no herniation of cerebellum. | Multifocal forebrain | Yes – intraparenchymal brain | NA | No | No |
| 65 | Fisher et al., 2024 | Multifocal CNS and PNS (myelopathy and neuropathy/myopathy) | Brain | Cerebellar atrophy, with decreased distinction between grey and white matter. It is surrounded by thick band of T2w and FLAIR hyperintense signal which is diffusely T1W hypointense, separating the folia of the cerebellum. Cerebral atrophy. No contrast enhancement.  A well-defined rounded shape T1w hyperintense structure is present within the posterior lobe of the pituitary gland. The structure is not visible in the T2w sequences as well as DWI, and homogeneously contrast enhancing.  Mild amount of T2w hyperintense signal, T1w hypointense, partially suppressed on FLAIR, along the roof of the nasopharynx.  Incidental T2w/T1w/FLAIR hyperintense, pedunculated structure, located at the left vertical external ear canal. | Diffuse cerebellar | No | Grey | No | Yes |
| 66 | Fisher et al., 2024 | Multifocal (forebrain and brainstem) | Brain | Cystic change on left ventral thalamus, intra-axial T2W and FLAIR periventricular and frontal lobe hyperintensities, no contrast enhancement.  Mild undulation of brain surface at longitudinal fissure (possible polymycrogyria). | Multifocal forebrain | No | NA | No | No |
| 67 | Fisher et al., 2024 | PNS  Neuromuscular | Spine | T2W focal mild hyperintensity at C6, mild intramedullary and ventral meningeal contrast enhancement, streaky enhancement of paraspinal muscles ventral to spine. | Focal spinal  Epaxial musculature | Yes – meningeal  Yes - intramuscular | NA | Yes | NA |
| 68 | Albertini et al., 2022 | Multifocal intracranial | Brain | Multifocal, diffuse, bilateral asymmetrical, ill-defined, intra-axial lesions affecting mainly the cortical grey matter, rather than white matter, and hippocampus. Focal, bilateral, asymmetrical, ill-defined intra-axial lesions affecting the caudate nucleus, thalamus, cerebellar nodulus, midbrain, medulla oblongata and cervical spinal cord were also noticed. The lesions were hyperintense in T2w and FLAIR images and iso- to hypointense in T1w images. The lesions were mildly to markedly diffusely contrast enhancing. | Multifocal/diffuse forebrain, brainstem, cerebellum | Yes – intraparenchymal brain | Grey | No | No |
| 69 | Lopes et al., 2022 | Multifocal (forebrain and cerebellum) | Brain | Cerebral and cerebellar atrophy. Ventriculomegaly. | Diffuse forebrain and cerebellum | No | Grey | No | No |
| 70 | Kennedy et al., 2024 | PNS - myopathy | Brain | Patchy increased T2W FSE & STIR hyperintensity of the right temporal and masseter musculature with contrast enhancement. No definite brain abnormalities. Unremarkable brain. | Masticatory muscles | Yes - intramuscular | None | Yes | No |
| 71 | Kennedy et al., 2024 | Multifocal | Brain | Severe T2W hyperintensity at the periphery of the cerebellum & multiple T2W hyperintensities within the cerebrum with varying degrees of contrast uptake, marked in the cerebellum. | Multifocal forebrain, cerebellum | Yes – intraparenchymal brain | NA | No | Yes - suspected |
| 72 | Kennedy et al., 2024 | Multifocal | Brain | Marked T2W hyperintensity of the white matter of the left cerebral hemisphere with midline shift to the right and areas of marked contrast enhancement. Marked muscle atrophy of the right masticatory musculature with patchy marked contrast enhancement. | Focal forebrain  Masticatory muscles | Yes – intraparenchymal brain  Yes - intramuscular | White | Yes | No |
| 73 | Kennedy et al., 2024 | PNS – neuropathy | Spine | Reduced SI in the epaxial muscles, T2W hyperintense striated appearance of thoracic and pelvic extremity musculature which is strongly contrast enhancing. | Epaxial musculature | Yes – intramuscular | NA | Yes | NA |
| 74 | Kennedy et al., 2024 | Cerebellum | Brain | Markedly increased T2W SI of the meninges of the cerebellum, highlighting the cerebellar gyri and increased sulcal width; focal area (5mm x 2mm) of increased T2W signal intensity of the left brainstem, just caudal to the cerebellar peduncle. | Multifocal brainstem, cerebellum | No | NA | No | Yes - suspected |
| 75 | Kennedy et al., 2024 | CNS – cervical spinal cord | Brain | Possible atrophy in the cerebellar vermis and relatively small cerebellar hemispheres with increased T2W signal of meninges. | Diffuse cerebellar | No | Grey | No | Yes - suspected |
| 76 | Kennedy et al., 2024 | Multifocal | Brain | Patchy T2W hyperintensity within the brainstem and within the cerebellum, strongly contrast enhancing. Increased contrast uptake of the left optic nerve. T2W hyperintensity at the rostral horn of the left ventricle. T2W hyperintensity within the cervical spine without contrast enhancement. Patchy T2W hyperintensities within the masticatory muscles with some contrast enhancement. | Multifocal brainstem, cerebellum, forebrain, spine  Masticatory muscles | Yes – intraparenchymal brain  Yes - intramuscular | NA | Yes | No |
| 77 | Kennedy et al., 2024 | PNS – neuropathy | Spine | Patchy to linear hyperintensity on STIR sequences throughout the epaxial musculature, hypoaxial or iliopsoas muscles, gluteal muscles, muscles of the proximal hindlimbs, muscles of the scapulae bilaterally with streaky contrast enhancement. Spinal cord and nerve roots were unremarkable. | Epaxial musculature | Yes – intramuscular | None | Yes | NA |
| 78 | Kennedy et al., 2024 | Multifocal | Brain | Ventriclomegaly, with slight asymmetry (left larger than right). Marked, asymmetric, increased SI in T2W images of the peripheral grey matter of the cerebrum with all lobes affected. Thalamus also affected. Marked T2W hyperintensity of the surface of the cerebellum and thinning of the cerebellar folia. | Multifocal/diffuse forebrain, cerebellum | No | Grey | No | Yes – suspected |
| 79 | Kennedy et al., 2024 | Multifocal | Brain | Abnormal SI involving the superficial portion of the cerebellar structures involving both the hemispheres and vermis. Contrast enhancement over the surface of the cerebellum and ventrally over the pons and brainstem. | Multifocal brainstem, cerebellum | Yes – meningeal  Yes – intraparenchymal brain | Grey | No | Yes - suspected |
| 80 | Kennedy et al., 2024 | Spinal cord segments caudal to L3 | Spine | Unremarkable. | None | No | None | No | NA |
| 81 | Kennedy et al., 2024 | Multifocal: CNS and PNS (myopathy) | Brain | Multifocal T2W and FLAIR reduced SI lesions (worse on left) throughout olfactory, frontal, temporal lobes of the forebrain and midbrain, mild ventriculomegaly and cortical atrophy. | Multifocal forebrain, brainstem | No | NA | NA | No |
| 82 | Kennedy et al., 2024 | Multifocal CNS | Brain | Mild T2W and FLAIR hyperintensity within the brain stem, mild contrast enhancement within the cerebellum. | Multifocal brainstem, cerebellum | Yes – intraparenchymal brain | NA | No | No |

Legend:

NA – not available

**Table S4. Therapy and outcomes**

| **Case No.** | **Author and Year of Publication** | **Therapy** | **Duration of Treatment (Months)** | **Outcome** | **Survival After Diagnosis of Neosporosis (Months)** | **Time of follow-up (months)** |
| --- | --- | --- | --- | --- | --- | --- |
| 1 | Gaitero et al., 2006 | Doxycycline  Clindamycin | 0.16 | Euthanasia due to neosporosis associated deterioration | 0.16 | 0.16 |
| 2 | Garosi et al., 2010 | Clindamycin  TMPS | NA | Improvement  Relapse | >7 | 7 |
| 3 | Garosi et al., 2010 | Clindamycin  TMPS | 3 | Euthanasia due to neosporosis associated deterioration  Relapse | 3 | 3 |
| 4 | Garosi et al., 2010 | Clindamycin  TMPS | 7 | Euthanasia due to neosporosis associated deterioration  Relapse | 7 | 7 |
| 5 | Garosi et al., 2010 | Clindamycin  TMPS | NA | Improvement | >7 | 7 |
| 6 | Garosi et al., 2010 | Clindamycin  TMPS | 2 | Remission | >6 | 6 |
| 7 | Garosi et al., 2010 | TMPS  Pyrimethamine | NA | Improvement | >7 | 7 |
| 8 | Garosi et al., 2010 | Prednisolone | 0.5 | Euthanasia due to neosporosis associated deterioration | 0.5 | 0.5 |
| 9 | Perzefall et al., 2014 | Clindamycin  TMPS | >8 | Improvement | >8 | 8 |
| 10 | Perzefall et al., 2014 | Clindamycin  TMPS  Pyrimethamine | >4 | Improvement  Relapse (after 4 months) | >4 | 4 |
| 11 | Perzefall et al., 2014 | Clindamycin  TMPS | >4 | Improvement | >4 | 4 |
| 12 | Perzefall et al., 2014 | Prednisolone  Clindamycin  TMPS | 5 | Improvement Died due to unrelated issue | 10 | 10 |
| 13 | Coelho et al., 2018 | Clindamycin  TMPS | 8 | NA | >8 | 8 |
| 14 | Coelho et al., 2018 | None | NA | NA | NA | NA |
| 15 | Coelho et al., 2018 | None | NA | NA | NA | NA |
| 16 | Coelho et al., 2018 | None | NA | NA | NA | NA |
| 17 | Coelho et al., 2018 | Prednisolone  Clindamycin  TMPS | NA | NA | NA | NA |
| 18 | Coelho et al., 2018 | Corticosteroids  Cytarabine  Clindamycin | NA | NA | NA | NA |
| 19 | Coelho et al., 2018 | TMPS  Pyrimethamine | NA | NA | NA | NA |
| 20 | Coelho et al., 2018 | Corticosteroids  Cytarabine | NA | NA | NA | NA |
| 21 | Coelho et al., 2018 | Clindamycin  TMPS | NA | NA | NA | NA |
| 22 | Didiano et al., 2020 | Clindamycin  TMPS | 7.5 | Remission | >9 | 9 |
| 23 | Didiano et al., 2020 | Clindamycin  TMPS | >3 | Improvement | >3 | 3 |
| 24 | Didiano et al., 2020 | Clindamycin  TMPS | >8 | Improvement  Euthanasia due to suspect unrelated issue | 8 | 8 |
| 25 | Didiano et al., 2020 | Clindamycin  TMPS | 6 | Improvement | >24 | 24 |
| 26 | Didiano et al., 2020 | Clindamycin  TMPS | 2.5 | Improvement | 24 | 24 |
| 27 | Didiano et al., 2020 | Clindamycin  TMPS  Pyrimethamine | 6 | Remission | >7 | 7 |
| 28 | Clark et al., 2022 | Clindamycin | 2 | Improvement | >2 | 2 |
| 29 | Clark et al., 2022 | Clindamycin  TMPS | 0.1 | Euthanasia due to neosporosis associated deterioration | 0.16 | 0.16 |
| 30 | Alf et al., 2024 | Prednisolone  Cyclosporine | 1 | Euthanasia due to neosporosis associated deterioration | 1 | 1 |
| 31 | Alf et al., 2024 | Prednisolone  TMPS | 2 | Euthanasia due to neosporosis associated deterioration | 2 | 2 |
| 32 | Alf et al., 2024 | Prednisolone | 0.4 | Euthanasia due to neosporosis associated deterioration | 0.4 | 0.4 |
| 33 | Fisher et al., 2024 | Clindamycin | NA | NA | NA | NA |
| 34 | Fisher et al., 2024 | Prednisolone  Clindamycin  TMPS | >6 | Improvement  Worsening due to suspected unrelated issue | >6 | 6 |
| 35 | Fisher et al., 2024 | Clindamycin | 1.5 | Improvement | >3 | 3 |
| 36 | Fisher et al., 2024 | Prednisolone  Clindamycin  TMPS | >3 | Improvement Relapse | >3 | 3 |
| 37 | Fisher et al., 2024 | Prednisolone  Clindamycin  Pyrimethamine  TMPS | >3 | Improvement | >3 | 3 |
| 38 | Fisher et al., 2024 | Prednisolone  Clindamycin | 1 | Remission | >3 | 3 |
| 39 | Fisher et al., 2024 | Prednisolone  Clindamycin  TMPS  Pyrimethamine | 4 | Improvement | >3 | 3 |
| 40 | Fisher et al., 2024 | Prednisolone  Clindamycin | 3 | Improvement, Relapse (after 26 months) | 26 | 26 |
| 41 | Fisher et al., 2024 | Prednisolone  Clindamycin | 0.13 | Euthanasia due to neosporosis associated deterioration | 0.13 | 0.13 |
| 42 | Fisher et al., 2024 | Prednisolone  Clindamycin  TMPS  Pyrimethamine | >6 | Improvement, Relapse (after 13 months) | >15 | 15 |
| 43 | Fisher et al., 2024 | Prednisolone  Clindamycin  TMPS | 2 | Improvement  Relapse (after 5.8 months) | >5.8 | 5.8 |
| 44 | Fisher et al., 2024 | None | 0 | Euthanasia before treatment | 0.03 | 0.03 |
| 45 | Fisher et al., 2024 | Clindamycin | 3 | Improvement  Relapsed twice (after 8.3 and at unknow time point) | 8.3 | 8.3 |
| 46 | Fisher et al., 2024 | Prednisolone  Clindamycin  Pyrimethamine | NA | Improvement  Relapse (after 16 months) | >16 | 16 |
| 47 | Fisher et al., 2024 | Prednisolone  Clindamycin  TMPS  Pyrimethamine | 3 | Improvement  Euthanasia due to unrelated issue | 3 | 3 |
| 48 | Fisher et al., 2024 | Prednisolone  Clindamycin  TMPS  Pyrimethamine | >6 | Improvement  Relapse (after 7 days)  Improvement  Euthanasia due to neosporosis associated deterioration | 30.6 | 30.6 |
| 49 | Fisher et al., 2024 | Prednisolone  Clindamycin  TMPS | 3 | Improvement  Relapse (after 2.3 months)  Improvement | 3 | 3 |
| 50 | Fisher et al., 2024 | Clindamycin | NA | Improvement  Lost to follow-up | NA | 0.25 |
| 51 | Fisher et al., 2024 | TMPS  Pyrimethamine | 4 | Improvement Relapsed (after 20.9 months) | 20.9 | 20.9 |
| 52 | Fisher et al., 2024 | Clindamycin | 1 | Improvement  Relapse (after 4.5 months) | 4.5 | 4.5 |
| 53 | Fisher et al., 2024 | Clindamycin | NA | Improvement  Euthanasia (due to poor QoL) | 26.6 | 26.6 |
| 54 | Fisher et al., 2024 | Prednisolone  Clindamycin  Pyrimethamine | 2 | Improvement | >3 | 3 |
| 55 | Fisher et al., 2024 | Prednisolone  Clindamycin | 0.25 | Euthanasia due to neosporosis associated deterioration | 0.25 | 0.25 |
| 56 | Fisher et al., 2024 | TMPS | 8 | Improvement  Euthanasia due unrelated issue | >8 | 8 |
| 57 | Fisher et al., 2024 | Prednisolone  Clindamycin  TMPS | 0.3 | Euthanasia due to neosporosis associated deterioration | 0.3 | 0.3 |
| 58 | Fisher et al., 2024 | Clindamycin | NA | NA | >3 | 3 |
| 59 | Fisher et al., 2024 | Prednisolone  Clindamycin  Pyrimethamine | NA | Improvement  Relapsed (at an unknown time point)  Euthanasia 45.7 months later due to unrelated issue | 45.7 | 45.7 |
| 60 | Fisher et al., 2024 | Clindamycin | NA | NA | NA | NA |
| 61 | Fisher et al., 2024 | Prednisolone  Clindamycin  TMPS | 11 | Remission | >11 | 11 |
| 62 | Fisher et al., 2024 | Prednisolone  Clindamycin  TMPS | >3 | Improvement | >3 | 3 |
| 63 | Fisher et al., 2024 | Clindamycin | NA | NA | NA | NA |
| 64 | Fisher et al., 2024 | Prednisolone  Clindamycin | 1.5 | Improvement, Euthanasia due to status epilepticus | 1.5 | 1.5 |
| 65 | Fisher et al., 2024 | Prednisolone  Clindamycin  TMPS | >10 | Improvement | >10 | 10 |
| 66 | Fisher et al., 2024 | Prednisolone  Clindamycin  TMPS | 2 | Improvement  Euthanasia 2 months later due to neosporosis associated deterioration | 2 | 2 |
| 67 | Fisher et al., 2024 | Prednisolone  Clindamycin  TMPS  Pyrimethamine | NA | NA | NA | NA |
| 68 | Albertini et al., 2022 | Dexamethasone  Prednisolone  Clindamycin  TMPS | 0.1 | Euthanasia due to neosporosis associated deterioration | 0.1 | 0.1 |
| 69 | Lopes et al., 2022 | None | 0 | Euthanasia on owner’s decision before treatment | 0 | 0 |
| 70 | Kennedy et al., 2024 | Clindamycin  Other medications NA | 0.8 | Poor improvement  Death due to neosporosis | 1.3 | 1.3 |
| 71 | Kennedy et al., 2024 | Clindamycin  Other medications NA | 3.9 | Remission | 34.3 | 34.3 |
| 72 | Kennedy et al., 2024 | Clindamycin  Other medications NA | NA | Improvement Multiple relapses | 38.5 | 38.5 |
| 73 | Kennedy et al., 2024 | Clindamycin  Other medications NA | 0.23 | Deterioration and death due to neosporosis | 0.23 | 0.23 |
| 74 | Kennedy et al., 2024 | Clindamycin  Other medications NA | NA | Improvement | 15.1 | 15.1 |
| 75 | Kennedy et al., 2024 | Clindamycin  Other medications NA | NA | Improvement  Euthanasia due to neosporosis | 2.3 | 2.3 |
| 76 | Kennedy et al., 2024 | Clindamycin  Other medications NA | 0.5 | Deterioration Euthanasia due to neosporosis | 0.5 | 0.5 |
| 77 | Kennedy et al., 2024 | Clindamycin  Other medications NA | NA | Improvement  Death due to unrelated issue | 60.8 | 60.8 |
| 78 | Kennedy et al., 2024 | Clindamycin  Other medications NA | 0.43 | Deterioration  Euthanasia due to neosporosis | 0.43 | 0.43 |
| 79 | Kennedy et al., 2024 | Clindamycin  Other medications NA | NA | Remission Multiple relapses,  Death due to unrelated issue | 132.4 | 132.4 |
| 80 | Kennedy et al., 2024 | Clindamycin  Other medications NA | 0.23 | Poor improvement Euthanasia due to neosporosis | 0.23 | 0.23 |
| 81 | Kennedy et al., 2024 | Clindamycin  Other medications NA | NA | Remission  Relapse (at unknown time point) Euthanasia due to neosporosis | 21.5 | 21.5 |
| 82 | Kennedy et al., 2024 | Clindamycin  Other medications NA | 0.43 | Poor improvement Euthanasia due to neosporosis | 0.43 | 0.43 |

Legend:

NA – not available

TMPS – trimethoprim sulfonamide
